# Biological Potential, Gastrointestinal Digestion, Absorption, and Bioavailability of Algae-Derived Compounds with Neuroprotective Activity: A Comprehensive Review

**DOI:** 10.3390/md20060362

**Published:** 2022-05-28

**Authors:** Bruna Martins, Mónica Vieira, Cristina Delerue-Matos, Clara Grosso, Cristina Soares

**Affiliations:** 1 LAQV, REQUIMTE, Instituto Superior de Engenharia do Porto, Instituto Politécnico do Porto, Rua Dr. António Bernardino de Almeida 431, 4249-015 Porto, Portugal; brunadaniela99@outlook.pt (B.M.); cmm@isep.ipp.pt (C.D.-M.); 2 Ciências Químicas e das Biomoléculas/CISA, Escola Superior de Saúde, Instituto Politécnico do Porto, Rua Dr. António Bernardino de Almeida 400, 4200-072 Porto, Portugal; mav@ess.ipp.pt

**Keywords:** Alzheimer’s disease, Huntington’s disease, neuroprotection, Parkinson’s disease, seaweeds

## Abstract

Currently, there is no known cure for neurodegenerative disease. However, the available therapies aim to manage some of the symptoms of the disease. Human neurodegenerative diseases are a heterogeneous group of illnesses characterized by progressive loss of neuronal cells and nervous system dysfunction related to several mechanisms such as protein aggregation, neuroinflammation, oxidative stress, and neurotransmission dysfunction. Neuroprotective compounds are essential in the prevention and management of neurodegenerative diseases. This review will focus on the neurodegeneration mechanisms and the compounds (proteins, polyunsaturated fatty acids (PUFAs), polysaccharides, carotenoids, phycobiliproteins, phenolic compounds, among others) present in seaweeds that have shown in vivo and in vitro neuroprotective activity. Additionally, it will cover the recent findings on the neuroprotective effects of bioactive compounds from macroalgae, with a focus on their biological potential and possible mechanism of action, including microbiota modulation. Furthermore, gastrointestinal digestion, absorption, and bioavailability will be discussed. Moreover, the clinical trials using seaweed-based drugs or extracts to treat neurodegenerative disorders will be presented, showing the real potential and limitations that a specific metabolite or extract may have as a new therapeutic agent considering the recent approval of a seaweed-based drug to treat Alzheimer’s disease.

## 1. Introduction

Marine organisms represent precious and unexplored resources of bioactive compounds with complex and unique structural features, and the interest in exploring their health-promoting effects is growing [1,2]. In the past decades, a large array of natural products with relevance in drug discovery have been isolated from bacteria, fungi, corals, micro and macroalgae, gorgonians, sponges, nudibranchs, bryozoans, sea cucumbers, tunicates, and sea hares, among other marine organisms [3].

Macroalgae can be classified into three broad groups based on their pigmentation: brown (Ochrophyta), red (Rhodophyta), and green (Chlorophyta) algae. These marine organisms are nutritionally rich, being a source of complex polysaccharides (especially fucoidan, laminarin, carrageenan), minerals (sodium, potassium, calcium, magnesium), proteins, vitamins (A, C, E, and those of the complex B), and mono and polyunsaturated fatty acids (docosahexaenoic acid (DHA), eicosapentaenoic acid (EPA)) as well as of several other phytochemicals [4,5]. Their consumption in western societies is only recent and had to be accompanied by marketing campaigns claiming their ‘superfood’ status and their ability to promote healthier lifestyles [6]. However, they have been consumed for centuries in Asian countries as part of the daily diet, with brown macroalgae being the most consumed (66.5%), followed by red (33%) and green (5%) algae [7].

Macroalgae bioactive metabolites are involved in several physiological functions, such as reproduction and growth. Under stress conditions (such as exposure to ultraviolet (UV) radiation, adverse temperature, salinity changes, and environmental pollution), seaweeds also produce a great variety of secondary metabolites, such as phenolic compounds, halogenated compounds, sterols, terpenes, and small peptides, among other bioactive compounds whose main function is their defence [8,9]. However, from a human perspective, the presence of these compounds contributes to the vision that macroalgae are functional foods and potential sources for drug development programs [10]. Regarding clinical trials, studies with seaweed metabolites are scarce, until recently restricted to those carried out with kahalalide F [11], fucoxanthin [12], and griffithsin [13], but recently other compounds were also tested for cognitive improvement in patients with neurodegenerative disorders [14,15]. 

It has been reported that crude extracts or purified components of macroalgae exhibit anticoagulant, antimicrobial, anticancer, antioxidant, antidiabetic, and antihypertensive activities, which might find relevance in cosmeceutical, nutraceutical, and pharmaceutical applications [16,17,18,19,20,21,22,23]. Furthermore, possible effects of macroalgae against neurodegenerative diseases have been studied [24,25,26,27,28,29].

It is estimated that more than 30 million individuals suffer from chronic or acute neurodegenerative disorders worldwide. Furthermore, given that life expectancy increases, more individuals will suffer from age-related diseases in the next few decades [30]. Alzheimer’s disease (AD) is the most prevalent neurodegenerative disease worldwide. In Europe, AD incidence is 19.4 per 1000 person-years in the population aged 65 and over [31].

Human neurodegenerative diseases are a heterogeneous group of illnesses [32] characterized by progressive loss of neuronal cells and nervous system dysfunction [33]. Neuroprotective compounds are essential in the prevention and management of neurodegenerative diseases. Antioxidant, anti-inflammatory, anti-excitotoxicity, enzyme inhibitors, anti-protein aggregation, and matrix metalloproteinase (MMP) inhibitor agents are examples of neuroprotective drugs [34,35,36,37]. 

Several reviews reporting the neuroprotective effects of macroalgae metabolites have been recently published. Barbalace et al. [38] reviewed the anti-inflammatory compounds isolated from seaweeds with potential effective protection against neuroinflammation. These authors highlighted the main inflammatory processes associated with neurodegeneration and the potential effect of the identified compounds from seaweeds that could reduce neuroinflammation in the central nervous system. In another review [39], the pathophysiology of neurodegenerative diseases and brain injuries were identified in order to determine the targets of pharmacological intervention focusing on the neuroprotective activities of seaweed compounds along with the underlying pharmacological mechanism, and the recent therapeutical advances. These authors also proposed a strategy to help the development of seaweed-based drugs [39]. Schepers et al. [40] focused their research particularly on the seaweed-derived phytosterols for the treatment of neurodegenerative disorders due to their characteristics: potentiate neuroplasticity, enhance phagocytic clearance of neurotoxic peptides, and have anti-inflammatory properties. Another recent review presented a critical overview of the seaweed secondary metabolites that revealed interesting results in in vivo and clinical studies [9].

Although a significant number of algal compounds with potential neuroprotective activity have been identified over the last decades, only a few were used in clinical trials related to neurodegeneration or cognitive impairments. However, the recent approval in China of sodium oligomannate, a marine algae-derived oligosaccharide for the treatment of AD, highlights the future of seaweed-based drug development [41].

This review will cover and discuss the last decade’s findings on the neuroprotective effects of bioactive compounds from macroalgae, highlighting their biological potential and possible mechanism of action, including microbiota modulation. Furthermore, the gastrointestinal digestion, absorption, and bioavailability of marine-derived neuroprotective compounds will be discussed. Moreover, the most recent clinical trials using seaweed-based drugs or extracts to treat neurodegenerative disorders will be presented, showing the real potential and shortcomings that a specific metabolite or extract may undergo as a new therapeutic agent.

### 1.1. Overview of Neurodegenerative Diseases

Neurodegeneration is characterized by a progressive and permanent loss of neurons in specified brain and spinal cord regions, which leads to a compromised motor or cognitive function [42]. It is the pathological condition that characterizes many neurodegenerative diseases, including AD, Parkinson’s disease (PD), and Amyotrophic lateral sclerosis (ALS), among others [43].

The neurodegeneration process involves cellular and molecular events such as abnormal protein misfolding and aggregation [44], neuroinflammation [45], oxidative stress [46], mitochondrial dysfunction [47], metal dyshomeostasis [48], and the reduction of neurotransmitter levels compromising the neurotransmission process [49].

#### 1.1.1. Protein Aggregates

Partial folding or misfolding turns protein functionally inactive because it can lead to self-association and subsequent deposition of the aggregated proteins [50]. This feature is common to several neurodegenerative disorders.

One of the hallmarks of AD is related to the extracellular deposition of β-amyloid (Aβ) peptides—the amyloid cascade hypothesis [51]. Aβ is produced from two sequential proteolytic cleavages of the Aβ precursor protein (APP), a transmembrane protein expressed by the APP gene, located in chromosome 21. APP is initially cleaved by either α-secretase or β-secretase. Both enzymes cleave the protein in the extracellular domain at different locations. In the α-secretase pathway, or non-amyloidogenic pathway, the enzyme is cut by the middle of the Aβ peptide producing a soluble protein (sAPPα), which has been reported to improve neurogenesis and cell survival [52]. In the amyloidogenic pathway, β-secretase also cleaves APP and produces a soluble protein (sAPPβ); however, the Aβ portion of APP is unaffected. The subsequent step in both pathways involves the cleavage of the internal portion of APP by γ-secretase, leading to the generation of the APP intracellular domain (AICD fragment), which is then translocated to the neuronal nucleus to regulate transcription [53,54]. Aβ peptides produced through the amyloidogenic pathway can have several lengths, between 38 and 42 amino acids [55], the most common forms being Aβ40 and Aβ42 [56]. The variation of Aβ lengths is related to the imprecise cleavage promoted by γ-secretase [55].

On the other hand, tau, a known microtubule-associated protein (MAP) codified by the MAPT gene located in chromosome 17, plays an important role in microtubule formation and maintaining the microtubules that form part of the neuron cytoskeleton. Tau contributes to neuronal stability; therefore, aberrant tau leads to neurodegeneration [53,57]. Hyperphosphorylation of tau is another feature of AD. In a healthy brain, tau phosphorylation is a well-balanced process between kinases and phosphatases. However, in some pathologies, such as AD, the phosphorylation process is disrupted. For example, glycogen synthase kinase 3 (GSK3) can phosphorylate tau at numerous phosphorylation sites, lending to the disbanding of microtubule-Tau bonds, thus reducing microtubule stability [58,59]. The hyperphosphorylated tau can aggregate into intracellular insoluble neurofibrillary tangles (NFTs), accumulating within the neurons of AD patients [60]. The primary toxic effects of NFTs involve the weakening of the microtubular structures and the disruption of neuronal transport, which can culminate in synapse loss and cell death [61].

α-Synuclein is a presynaptic protein found in axonic terminals of dopaminergic neurons and is encoded by the SNAC gene. α-Synuclein seems to regulate neuronal physiological processes by interacting with the lipid membrane or its anchored proteins, contributing to vesicle trafficking, vesicle fusion with the lipidic membrane, and neurotransmitter release [62,63]. Lewy bodies and Lewy neurites are typical hallmarks of PD and are composed of misfolded α-synuclein [59]. Intracellular aggregation and deposition of α-synuclein into insoluble inclusions is toxic and can lead to dysfunction of the dopaminergic neurons [64]. The abnormal aggregation of unfolded α-synuclein oligomers can lead to the formation of larger, toxic agglomerates, such as protofibrils and fibrils. Fibrils are much more stable than oligomers (and protofibrils), suggesting that the latter species are more neurotoxic, as some studies evidenced a poor correlation between the size of Lewy bodies with the severeness of PD effects [65,66].

HD is one of the polyglutamine-related neurodegenerative diseases [67]. HD is caused by a mutation in the huntingtin (HTT) gene on chromosome 4 that abnormally expands the number of CAG nucleotide repeats [68]. The wild-type huntingtin gene contains a CAG repeat in the range of 6 to 35, but in HD, 36 or more repeats occur [69]. The protein resulting from polyglutamine expansion—HTT—aggregates into protein deposits—the inclusion bodies (IBs)—and the longer the CAG repeat, the more toxic the generated fragment [70].

ALS is a fatal disease involving the central (CNS) and peripheral (PNS) nervous systems compromising the motor function [71]. ALS can be familial or sporadic, the sporadic form being more common. Both forms of this disease are characterized by cytoplasmic protein aggregates accumulation typically formed by misfolded proteins, including superoxide dismutase 1 (SOD1), TAR DNA-binding protein 43 (TDP-43), fused in sarcoma (FUS), optineurin (OPTN), ubiquilin 2 (UBQLN2), and chromosome 9 open reading frame 72 (C9orf72) dipeptide repeat (DPR). These protein aggregates occur in motor neurons and surrounding oligodendrocytes [72].

The protein aggregates and depositions are the trigger factors for several neurotoxic pathways, including excitotoxicity, oxidative stress, inflammation, and neuronal death [51,73]. Autophagy regulates the clearance of aggregated proteins that can cause several neurodegenerative disorders such as HD [74], PD [75], and AD [76]. The loss of basal autophagy in mouse neuronal cells achieved by knockdown of the essential autophagy genes Atg5 or Atg7 (autophagy-related 5 and 7) results in cytoplasmic aggregate accumulation and leading to neurodegeneration [77].

#### 1.1.2. Oxidative and Nitrosative Stress

The brain is very susceptible to oxidative stress because it is an active tissue that depends on a large amount of energy produced by oxidative phosphorylation and has a high density of oxidizable substrates (lipids) and a relative deficiency in antioxidant systems [78,79]. The cause/effect relationship between oxidative stress and protein aggregates characteristic of AD, PD, and HD has been hypothesized. Regarding PD pathology, it is reported that oxidative stress promotes α-synuclein aggregation in dopaminergic neurons and that α-synuclein further generates intracellular ROS [80,81]. Moreover, free radicals are also implicated in misfolding and accumulation of mHtt [82], and Aβ-mediated ROS production was reported to induce oxidative damage on both the Aβ peptide and surrounding molecules [83,84].

Oxidative phosphorylation occurs in mitochondria, and reactive oxygen species (ROS) can be generated during mitochondrial oxidative metabolism and due to environmental conditions such as pollution, radiation, and smoking [85,86]. ROS include the superoxide anion radical (O_2_^●−^), hydrogen peroxide (H_2_O_2_), hydroxyl radical (^●^OH), singlet oxygen (^1^O_2_), peroxyl radical (LOO^●^), alkoxyl radical (LO^●^), lipid hydroperoxide (LOOH), hypochlorous acid (HOCl), peroxynitrite (ONOO^−^), and ozone (O_3_), among others [87].

Cells typically have mechanisms to neutralize the damage induced by free radicals. These mechanisms include enzymatic antioxidants such as SOD, catalase (CAT), and glutathione peroxidase (GPx) and non-enzymatic antioxidants such as glutathione (GSH) and vitamins C and E [88,89]. ROS are considered essential for neuronal development and function in moderate or low amounts. However, when ROS levels overwhelm antioxidant systems, oxidative stress occurs. This situation can lead to extensive protein oxidation and lipid peroxidation, causing oxidative damage and cellular degeneration and is involved in acute and chronic CNS injury [90,91]. Excessive ROS production can be caused by mitochondrial dysfunction or inflammation [92].

Additionally, protein aggregates contribute to the appearance or/and increase oxidative stress; for example, Aβ aggregation can activate the pro-oxidative enzyme nicotinamide adenine dinucleotide phosphate (NADPH) oxidase [93].

Moreover, nitrosative stress is also a hallmark of neurodegeneration. It is provoked by increased production of reactive nitrogen species (RNS) such as nitric oxide (^●^NO), peroxynitrite (ONOO^−^), and nitrogen dioxide radical (NO_2_^●^) [87]. Several nitrogen species, including ^●^NO and peroxynitrite, are overproduced during the inflammatory process [94]. In addition, ^●^NO can react with O_2_^●−^ to form ONOO^−^ which can further convert to highly toxic intermediates such as NO_2_^●^, ^●^OH, and carbonate radicals [95].

#### 1.1.3. Metal Dyshomeostasis

Metals are a large group of compounds that can have different effects on the human organism. For example, lead (Pb) and aluminium (Al) can be toxic metals, depending on the degree of exposure. At the same time, zinc (Zn), iron (Fe), and copper (Cu), at low concentrations, are considered essential for some biological functions [96,97,98]. They are essential for regulating cellular pathways that are fundamental for brain function, such as neurotransmitter synthesis and release, neurotransmission, and protein turnover [48].

Brain metal accumulation increases with age [99,100]. A recent meta-analysis revealed a reduction in Cu levels and increased Fe levels in postmortem substantia nigra tissue from PD patients [101]. In addition, meta-analysis studies also revealed that brain Fe levels increase in AD patients [102] and serum Cu levels are also slightly enhanced [103]. Dyshomeostasis of these transition metals is related to neurodegeneration, involving oxidative stress and protein misfolding, as reviewed previously [104,105,106]. Metal dyshomeostasis can lead to the abnormal functioning of the ubiquitin—proteasome system (UPS), a crucial protein degradation system in eukaryotes. Zn^2+^ and Cu^2+^ can inhibit self-polyubiquitination reactions [107]. Metal chelators can be one strategy to control the dysregulated content of metal ions, as reported in several studies [108,109].

Aβ interactions with Zn^2+^, Cu^2+^, and Fe^3+^ have been reported [110,111,112]. These metals are co-localized with Aβ peptides and may be involved in their formation, being found at high concentrations—Cu at 400 mM, Zn at 1 mM, and Fe at 1 mM [113]. These metals associated with Aβ peptide can be reduced, consequently causing overconsumption and depletion of endogenous antioxidants in neurons [109,114,115].

The exposure of SH-SY5Y cells to Fe^3+^ increased β-secretase activity and production of Aβ_1–42_ [116]. In addition, the exposure of Fe to male APP/PS1 transgenic mice induced cyclin-dependent kinase 5 (CDK5) and glycogen synthase kinase 3β (GSK3β) activity, leading to tau phosphorylation [117].

In silico analysis of genes involved in brain metal homeostasis revealed a decreased expression of copper-dependent enzymes such as COX, SOD1, and a copper-chaperone protein (ATOX1) in AD brains. On the other hand, metallothioneins, a family of small, highly conserved, cysteine-rich metal-binding proteins, are overexpressed in AD and PD, along with Fe binding ferritin in AD and Fe binding transferrin in PD [118].

Transferrin is a reversible chelator that can bind two atoms of ferric iron (Fe^3+^), and the dissociation can occur in an acidic environment such as in endosomes. This process can maintain Fe^3+^ in a soluble form under physiological conditions, regulate iron transport and cellular uptake more easily, and maintain Fe^3+^ in a redox-inert state [119].

Ceruloplasmin produced in the liver is a major Cu binding glycoprotein in blood plasma and is also present in cerebrospinal fluids, among others. The ceruloplasmin exerts several functions, including Cu transport, regulation of Fe homeostasis, and ferroxidase activity [120,121]. Excess Cu is chelated by metallothioneins and glutathione to prevent redox activity and ROS production [48]. In multiple sclerosis patients, Cu levels in cerebrospinal fluid are increased, possibly by reducing the ferroxidase function of serum ceruloplasmin, due to the oxidative environment in serum [122]. Similarly, the ferroxidase ceruloplasmin is oxidized and deamidated in the oxidative environment of the cerebrospinal fluid in PD patients [123].

Most plasma zinc ions are bonded with albumin and α2-macroglobulin, which serve as a zinc pool in circulation. Zinc homeostasis in the brain is strictly regulated, mainly by three families of proteins: metallothioneins, zinc- and iron-like regulatory proteins, and zinc transporters [124].

#### 1.1.4. Neuroinflammation

Neuroinflammation is a defence mechanism that initially protects the brain by promoting tissue repair and removing cellular debris. However, sustained inflammatory responses are detrimental and inhibit regeneration. Neuroinflammation can be triggered by several factors such as CNS infection, trauma, exposure to environmental factors, tumours, toxins, and age that can activate microglia. Microglia are also activated against protein aggregates such as Aβ, α-synuclein, mutant htt, and mutant or oxidized SOD [125,126,127,128]. The interactions between Aβ and microglia occur through microglial pattern recognition receptors (PRRs) such as Toll-like receptors (TLR2, TLR4, TLR6, and TLR9) and complement receptor 3 (CR3). These interactions activate microglia leading to the production of pro-inflammatory cytokines and chemokines. Microglia triggers Aβ fibrils phagocytosis, while soluble Aβ can be degraded by extracellular proteases such as neprilysin and insulin-degrading enzyme (IDE) [128,129].

Microglia are considered the principal resident innate immune cells of the CNS and regulate several processes during inflammation, such as cell death and elimination of waste products. In addition, they are involved in the refinement of synaptic networks, promoting developmental apoptosis and removing apoptotic cell corpses, positioning of neurons within developing barrel cortex, precise secretion of growth factors for neuronal survival, and producing neuromodulatory factors that support synaptic plasticity and learning [130].

Excessive glial cell activation has a role in pro-inflammatory action and can lead to synaptic dysfunction, loss of synapses, and neuronal death resulting in neurodegeneration [38]. Activated microglial leads to overproduction of pro-inflammatory mediators, such as interleukins (IL) IL-1β, IL-2, IL-6, tumour necrosis factor-α (TNF-α), inducible nitric oxide synthase (iNOS), cyclooxygenase 2 (COX2), ROS/RNS, as well as matrix metalloproteinase (MMPs) and chemokines [125,131,132,133]. These factors and enzymes are up-regulated by several intracellular pathways that involve signal transduction molecules such as nuclear factor-kappa B (NF-κB), mitogen-activated protein kinases (MAPKs), signal transducer and activator of transcription (STAT), and phosphatidylinositol-3 kinase (PI3K) [126,134]. The transcriptional activation of NF-κB can be triggered by ROS, such as H_2_O_2_ [135]. The PI3K/Akt cascade is activated by cytokines, LPS, and growth factors [134,136].

Reactive microglia have been established to closely colocalize with Aβ plaques in the brains of patients with AD [137,138,139] and have also been found near NFT-bearing neurons [140,141]. Moreover, it was shown that aggregated α-synuclein activates microglia, leading to proinflammatory mediators in PD brains [142]. Activated microglia also appeared in HD brains indicating that mutant htt aggregates stimulate microglia activation [143].

#### 1.1.5. Mitochondrial Dysfunction

Mitochondria is an organelle that regulates cell metabolism, intracellular calcium (Ca^2+^) homeostasis, and apoptosis [144]. Various transcription factors, including nuclear respiratory factors 1 and 2 (NRF1 and NRF2), cAMP response element-binding protein 1 (CREB1), peroxisome proliferator-activated receptors δ (PPAR δ), and others, are involved in the generation of new functional mitochondria [144]. Repetitive mitochondrial fusion and fission cycles and the balance between fusion and fission are crucial for mitochondrial morphology and, consequently, mitochondrial dynamics. Failure in this balance can increase lipid peroxidation, decrease membrane potential, decrease ATP production, and compromise organelles’ inheritance during cytokinesis [145,146]. Dysfunctional mitochondria and, therefore, loss of energy production are important contributors to the pathophysiology associated with neurodegenerative disease [50].

Mitophagy is an autophagic breakdown in which the phagophore recognizes dysfunctional and impaired mitochondria, re-directing them to be degraded by lysosomes. Failure in mitophagy can lead to the accumulation of dysfunctional mitochondria contributing to the progress of age-related diseases [145,147,148].

Neurotoxic protein aggregates cause impairment of mitochondrial integrity [145]. In addition, protein aggregates such as Aβ oligomers, NFTs, mutated htt fragments, and α-synuclein change gene expression in Ca^2+^ homeostasis, contributing to Ca^2+^ dyshomeostasis [149].

The excessive levels of Ca^2+^ in mitochondria can lead to mitochondrial swelling, loss of membrane potential, and thus cell death [149,150]. Additionally, excessive levels of Ca^2+^ in cytosol promote excitotoxicity that can be characterized by excessive stimulation of ionotropic glutamate receptors, such as the N-methyl-D-aspartate receptor (NMDAR) [151]. Abnormal NMDAR activity is associated with neurodegenerative disorders, such as AD [152], HD [153], and PD [154]. Indeed, memantine, one of the current drugs approved against AD, is an NMDAR antagonist [155].

#### 1.1.6. Neurotransmitters

Apart from the involvement of glutamate in neurodegeneration processes, the levels of other neurotransmitters are also affected.

Current therapies to treat AD patients are based on the “cholinergic hypothesis” consisting of cholinesterase enzyme inhibition—acetylcholinesterase (AChE) and butyrylcholinesterase (BChE). These enzymes cleave the neurotransmitter acetylcholine (ACh) [156]. The currently available commercial cholinesterase inhibitors are donepezil, galantamine, and rivastigmine. Donepezil and galantamine are reversible AChE inhibitors, while rivastigmine inhibits both cholinesterases [157,158]. ACh plays an essential role in memory. The postmortem brain samples of AD patients revealed a significant depletion in the cerebral cortex of choline acetyltransferase, the enzyme involved in ACh production [159]. However, the activity of some forms of cholinesterase is increased [160,161].

Dopamine plays a central role in pleasurable reward behaviour, mood, attention, learning, and behaviour and plays a crucial role in neuronal proliferation and differentiation in the adult CNS [162]. In PD, the levels of dopamine are low due to the loss of dopaminergic neurons in the substantia nigra, and therefore inhibition of the enzyme that metabolizes dopamine is one of the targets to treat this pathology [163]. Monoamine oxidases (MAOs) are responsible for the catabolism of monoamine neurotransmitters [164]. There are two isoforms, MAO-A and MAO-B. MAO-A selectively metabolizes dopamine, serotonin, and norepinephrine, while MAO-B catabolizes dopamine [165]. Therefore, MAO-B inhibitors are potential therapeutic agents for PD since they provide stable dopamine levels in the synaptic cleft, improving motor function [166]. The current MAO-B inhibitors available are selegiline, rasagiline, and safinamide, with safinamide being a reversible MAO-B inhibitor, while the others are irreversible inhibitors. Among all antiparkinsonian agents, MAO-B inhibitors have the greatest neuroprotective potential because of dopamine metabolism inhibition, induction of neurotrophic factors, and, in the case of safinamide, inhibition of glutamate release [167].

#### 1.1.7. Neurotransmitter Receptors and Other Straightly Related to Neurodegenerative Diseases

Dopamine receptors are involved in locomotion, motor learning, cognition, learning and memory, decision making, attention, impulse control, sleep, regulation of renal function, reproductive behaviour, renin secretion, and food intake [168]. There are five subtypes of dopamine receptors (D1–D5) members of the G-protein coupled receptor family. D1 receptors are the most abundant receptors in the human nervous system, followed by D2 receptors [162,168]. With ageing, the dopaminergic system declines, and D1 and D2 are the most affected receptors [169].

One meta-analysis revealed that D1 and D2 receptor levels decreased in patients with AD [170]. The stimulation of D2, D3, and D4 receptors decreases the release of ACh onto striatal cholinergic interneurons, while stimulation of D1 and D5 receptors increases the release of ACh [171,172]. In the case of PD, the density of striatal postsynaptic D1 and D2 receptors is increased in PD brains; they are counterbalanced with levodopa therapy, which induces the down-regulation of D1 and D2 receptors to normal densities [173,174]. Currently, dopamine receptor agonists are the first choice in de novo and young PD patient therapy or in combination with levodopa to retard the development of motor complications in advanced stages of PD. DA receptor agonists appear to act by direct stimulation of postsynaptic DA receptors and presynaptic receptors [173].

An imbalance between excitatory and inhibitory neurotransmission occurs in neurodegeneration. The leading players in this balance are glutamate, the major excitatory neurotransmitter, and gamma-aminobutyric acid (GABA), the major inhibitory neurotransmitter in CNS. Glutamate plays an essential role in memory, synaptic plasticity, and neuronal development. However, glutamate overstimulation is implicated in neurodegeneration, as explained above [175,176].

Glutamate receptors belong to two different groups: ionotropic and metabotropic receptor types. There are eight metabotropic glutamate receptors (mGluRs) [177]. Overstimulation of mGluRs in microglia and astrocytes can lead to a pro-inflammatory response being potentially able to induce neuronal death. To modulate the neuroinflammatory response mediated by these receptors, the negative allosteric modulation of mGlu2 and mGlu5 and positive allosteric modulation of mGlu3 can be applied [178,179].

Relative to ionotropic glutamate receptors (iGluRs), excitatory neurotransmission is tightly mediated by NMDA and α-amino-3-hydroxy-5-methylisoxazole-4-propionate (AMPA) and kainic acid (KA) receptors. Continuous activation of large numbers of NMDARs leads to increased intracellular Ca^2+^ and catabolic enzyme activities, which can trigger a cascade of events (mitochondrial membrane depolarization, caspase activation, production of toxic ROS/RNS, and cellular toxicity), ultimately leading to apoptosis or necrosis. AMPA receptors have also been implicated in excitotoxicity because they are highly permeable to Ca^2+^ [51,180]. The same events are triggered by kainic acid [181].

GABAergic receptors can be ionotropic (GABA_A_) or metabotropic (GABA_B_). GABA_A_ receptors mediate the rapid synaptic inhibition, while GABA_B_ receptors mediate the slow and prolonged activity. GABA_B_ exerts activity in both pre-synaptic and post-synaptic, inhibiting the release of neurotransmitters and mediating the hyperpolarization of the neuron, respectively [182].

GABA is involved in several physiological functions maintained through a complex interaction between GABA and Ca^2+^-dependent neurotransmission. For example, the glia cell-derived neurotrophic factor (GDNF) is controlled by the Ca^2+^/GABA; GDNF enhances the survival and function of the dopaminergic neurons in the midbrain. Conversely, failure to control Ca^2+^/GABA leads to the accumulation of intracellular Ca^2+^ deposits, thus contributing to neurodegeneration [183].

In the PD brain, the loss of nigrostriatal dopaminergic neurons is associated with a downregulation of the GABAergic tone and a prevalence of the excitatory system in the substantia nigra and basal ganglia. mRNA levels of GABA_A_ subunits α4 and β1 are reduced in substantia nigra, while the α4 subunit is increased about 22-fold in caudate nucleus regions. Increases in the GABA_A_ receptor also induce increased tonic inhibition by astrocytes [138,184]. Changes are also visible in AD brains. Protein or mRNA for GABA_A_ subunits α1, α5, and β3 decrease in the hippocampus of AD patients with severe neuropathology, and in the prefrontal cortex, a reduced amount of α1 and α2 subunit expression in early and late AD stages and downregulation of subunits α4, β2, and δ during the late stages of AD was observed [184,185].

Tyrosine kinase receptors (Trks) can be categorized into three groups (A, B, and C) based on their activation. TrkA is activated by the nerve growth factor (NGF), TrkB is activated by the brain-derived neurotrophic factor (BDNF) and neurotrophin 4 (NT4), and TrkC is activated by neurotrophin 3 (NT3) [186]. TrkB signalling is crucial for neuronal functions such as neuronal development and modulation of short- and long-lasting synaptic interactions. In addition, it regulates survival by controlling the Ras-PI3K-Akt signalling cascade [187,188]. Recent studies indicate that BDNF/TrkB signalling is decreased in several neurodegenerative diseases, including AD [189], PD [190,191], and HD [192], in both animal models and humans.

Toll-like receptors (TLRs) are implicated in inflammation response because TLRs are transmembrane pattern-recognition receptors (PRRs) that respond to pathogen-associated molecular patterns (PAMPs). TLRs can be classified based on the specific PAMPs they recognize. For instance, TLR4 recognizes LPS [193]. Several TLRs are expressed in microglia and neurons [193,194].

TLR activation leads to inflammation and induces pro-inflammatory cytokine release. In AD, TLR4 and TLR2 activation can be beneficial or detrimental, i.e., beneficial by increasing Aβ clearance while detrimental by inducing the production of pro-inflammatory cytokines [195]. Aggregated forms of α-synuclein can act as ligands for TLRs, mainly TLR2 and TLR4, which have been reported to be upregulated in many different PD animal models. Evidence from human studies further points to the implication of these receptors in the pathogenesis of PD [196] and HD [197].

## 2. Methods

The research was carried out in Google Scholar, Wiley, Pubmed, and Taylor & Francis databases.

Search terms were combined in different manners and included: macroalgae, neurodegenerative diseases, receptors, neurotransmitters, protein aggregates, enzyme inhibitors, antagonists, agonists, Alzheimer’s disease, Parkinson’s disease, Huntington’s disease, polysaccharides, fucoidan, protein hydrolysates, bioactive peptides, PUFAs, carotenoids, fucoxanthin, astaxanthin, β-carotene, phycobiliproteins, chlorophylls, phlorotannins, kaempferol, quercetin, biochanin A, epicatechin, epigallocatechin gallate, gallic acid, ferulic acid, caffeic acid, fucosterol, glycoprotein, glycolipid, bioaccessibility, bioavailability, microbiome.

**Figure 1:**
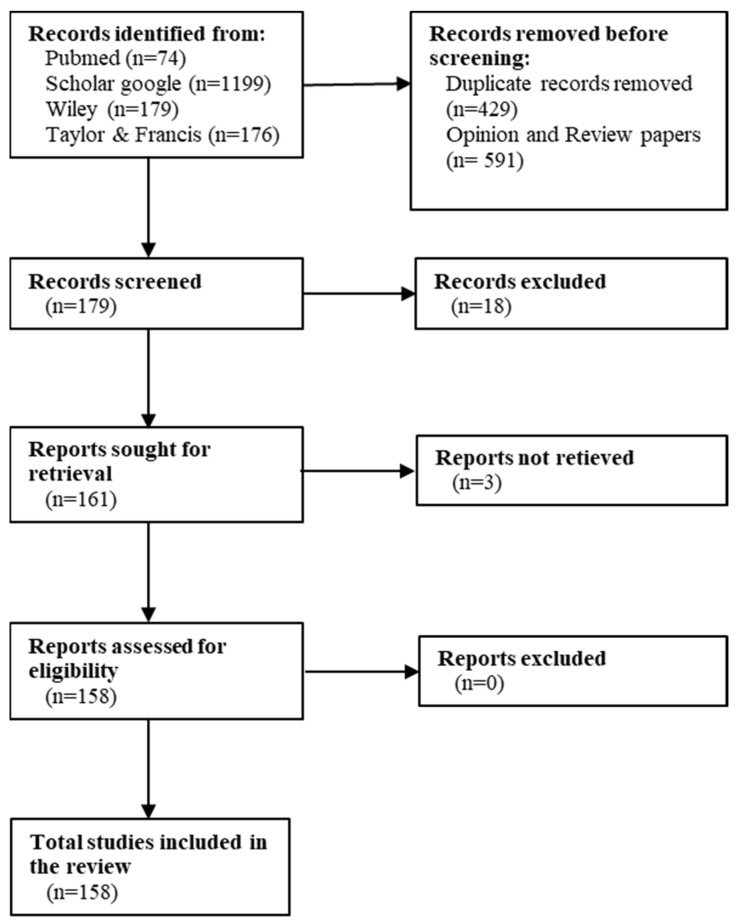
PRISMA flow diagram representing the original papers included in Section 3, Section 4, Section 5, Section 6 and Section 7.

Articles were selected based on two criteria: (1) articles that specifically address the neuroprotective effects of compounds derived from macroalgae that have been published in the last decade (2010–2022), and (2) only articles written in English (Figure 1).

## 3. Compounds with Potential Neuroprotective Activity Extracted from Macroalgae

The neuroprotective activity of seaweeds is related to several classes of compounds such as polysaccharides, proteins, lipids, pigments, or polyphenols. The total polysaccharide concentrations vary significantly among the seaweed species (4–76% DW) [198]. The protein content of Rhodophyta and Chlorophyta range in the 10–47%, while Ochrophyta varies 3–16% DW [199]. Relative to PUFAs, Rhodophyta and Ochrophyta have a high content of PUFAs [200]. Carotenoids are prominent in Ochrophyta species because fucoxanthin is an abundant carotenoid in seaweeds. Fucoxanthin represents 96%, 52%, and 49% of total carotenoids extracted from *Himanthalia elongata* (L.) S.F. Gray, *Laminaria ochroleuca* Bachelot Pylaie, and *Undaria pinnatifida* (Harvey) Suringar, respectively [201]. Finally, Ochrophyta has relevant phenolic compounds and has higher active antioxidants than Rhodophyta and Chlorophyta [8,202].

Table 1 displays the IC_50_ values for cholinesterases, β-site APP cleaving enzyme 1 (BACE1) and MAO inhibitions related to a given seaweed compound. These neuroprotection strategies are essential for AD, PD, and depression treatment. Currently, there are no commercially available drugs acting as BACE 1 inhibitors.

### 3.1. Polysaccharides

Polysaccharides are found in the cell walls of the macroalgae, contributing to physically supporting the thallus in water [214]. Polysaccharides represent about 40% of the dry weight (dw) in edible seaweeds [215]. The great variety of polysaccharides arises from the different monosaccharide constituents, degree of polymerization, sequence of sugar residues [216], and the presence of non-carbohydrate substituents such as positive and negative charged groups [217].

Polysaccharides from macroalgae include polyuronides, and some are methylated, acetylated, and sulfated [218]. The seaweeds are rich in sulfated polysaccharides (SPs), with fucoidan being found in brown algae, carrageenan in red algae, and ulvan in green algae. SPs are well recognized for their antioxidant activity. The presence of the sulfate group in their structure enhances antioxidant activity compared to non-sulfated polysaccharides, contributing to preventing cancer, diabetes mellitus, and neurodegenerative disease [216,219]. The sulfate group acts as an electrophile and promotes intramolecular hydrogen abstraction [220]. Relatively to their molecular weight, the polysaccharides with low molecular weight display enhanced antioxidant activity compared to that of high molecular weight because they can readily be incorporated into the cells and donate protons [215,221]. Recently, fucoidan biological activities from *Fucus vesiculosus* L. have shown to be a potential treatment against the COVID-19 virus [222] and for the development of new foods and drugs [223].

SPs at 0.8 mg/mL isolated from *Ecklonia maxima* (Osbeck) Papenfuss, *Gelidium pristoides* (Turner) Kützing, *Ulva lactuca* (Turner) Kützing Platboom, and *Gracilaria gracilis* (Stackhouse) Steentoft, L.M. Irvine & Farnham revealed neuroprotective effects against Zn (50 μM) in rats hippocampal neuronal cells (HT-22 cell line). The SPs isolated from *E. maxima* and *G. pristoides* improved cell viability, preventing late apoptosis and necrosis. SPs from all species also induced increased catalase and SOD activities, increased glutathione content, decreased ^●^NO level, and reduced AChE activity, counteracting the pro-oxidant and cholinergic impairment effects caused by Zn treatment [224].

Heteropolysaccharides rich in fucose, uronic acid and sulfate extracted from *Sargassum naozhouense* Tseng & Lu, *Sargassum thunbergii* (Mertens ex Roth) Kuntze, *Sargassum integerrimum* Tseng & Lu, and *Sargassum fusiforme* (Harvey) Setchell showed neuroprotective and antioxidant activities in MES 23.5 cells treated with 1 mg/mL of 6-hydroxydopamine (6-OHDA)—a neurotoxin widely used to induce models of PD. In addition, heteropolysaccharides extracted from *S. thunbergii* displayed ^●^OH and 1-1-diphenyl-2-picrylhydrazine (DPPH^●^) radical scavenging activities and reducing power; those from *S. fusiforme* and *S. integerrimum* only demonstrated DPPH^●^ scavenging activity and reducing power, and finally, *S. naozhouense* heteropolysaccharides were able to scavenge ^●^OH [225].

The structure of fucoidans from brown seaweeds is heterogeneous, consisting mainly of sulfated fucose and can have minor proportions of other sugars, such as xylose, uronic acids, and galactose [219].

1-Methyl-4-phenyl-1,2,3,6-tetrahydropyridine (MPTP) is commonly used to generate experimental models of PD. MPTP is converted into a neurotoxin, 1-methyl-4-phenyl pyridine (MPP^+^), that accumulates in dopaminergic neurons and causes inhibition of mitochondria complex I, thus impairing the respiratory chain [147,151]. Fucoidan (100 µM), whose composition consists mainly of L-fucose-4-sulfate, with an average molecular weight of 189 KD, attenuated the damage in dopaminergic nerve precursor cell line (MN9D) induced by 100 µM MPP^+^ by reducing the expression of light chain 3-II (LC3-II) and inhibition of the expression of cathepsin D (Cat D)-Bax [226]. LC3-II is an autophagosome membrane-bound protein and acts as an autophagy marker [227]. In addition, Cat D-Bax is associated with the apoptosis process since the overexpression of Cat-D activates Bax, a pro-apoptotic protein [228]. Hu et al. [229] revealed that a fucoidan (SFPS65A) isolated from the ethanol precipitation of *S. fusiforme*, at 250 mg/kg, enhanced the cognitive ability in male ICR mice treated with scopolamine, compared to a heteropolysaccharide SFPS65B extracted from the same species of macroalgae. SFPS65A, with 90 kDa, is composed of fucose, galactose, xylose, glucose, glucuronic acid, and mannose in the ratio of 19.23:9.58:6.64:1:6.52:2.57. It is a highly sulfated galactofucan compared to the less sulfated heteropolysaccharide SFPS65B [229].

A fucoidan (48% total sugar content, 28% fucose content, and 29% sulfate content) isolated from *Laminaria japonica* Areschoug was revealed to improve mitochondrial respiratory function by upregulation of peroxisome proliferator-activated receptor-gamma coactivator 1-α (PGC-1α)/NRF2 pathway, in a rat model of PD induced by rotenone [230]. The expression of PGC-1α and NRF2 contributes to mitochondrial biogenesis, NFR2 being responsible for activating numerous nuclear genes that contribute to mitochondrial respiratory function [231]. Similarly, crude fucoidan isolated from *F. vesiculosus* can upregulate the 5′ adenosine monophosphate-activated protein kinase (AMPK)-PGC-1α axis in a PD cell model induced by MPP^+^ [232] (Table 2). AMPK is a serine/threonine-protein kinase, and its activation is crucial for energy production and regulation of mitochondria biogenesis [233].

A purified fucoidan from *F. vesiculosus* can also ameliorate neuroinflammation by regulating several molecular pathways. This SP is shown to inhibit the activation of NF-кB, protein kinase-B (Akt), extracellular signal-regulated kinase (ERK), p38 MAPK, and c-Jun N-terminal kinase (JNK) in LPS-induced BV2 microglial cells. In addition, fucoidan has been shown to inhibit PGE2 production in a concentration-dependent manner [234] (Table 2). Although these molecular pathways are involved in inflammation and apoptosis, p38 MAPK and JNK are pro-apoptotic pathways [235].

In addition, fucoidan has been shown to have neuroprotective effects against protein aggregates. Fucoidans isolated from *F. vesiculosus* and *U. pinnatifida*, tested in the range of 3.125 to 100 μg/mL, revealed activity against Aβ_1–42_ aggregation in neuronal PC-12 cells [236] (Table 2). Wang et al. [237] reported that a commercially available fucoidan, at 100–500 ng/mL, decreased the Aβ accumulation in transgenic AD *Caenorhabditis elegans* by promoting proteasome activity and consequently reducing Aβ accumulation and alleviating oxidative stress [237].

In a recent study, a fucoidan (41.48% carbohydrate, 12.69% sulfates, and 13.90% uronic acid) showed potential neuroprotective effects against apoptosis induced by Aβ and D-galactose (D-Gal) in PC12 cells through caspase inhibition [238]. In the same study, fucoidan at 100 and 200 mg/kg revealed neuroprotective effects against D-Gal-induced learning and memory impairment in AD model mice by decreasing AChE activity and increasing the choline acetyltransferase activity [238]. Pretreatment with fucoidan at 50 mg/kg, intraperitoneally administered once daily for 5 days before transient global cerebral ischemia in a gerbil model, attenuated the loss of pyramidal neurons in the hippocampal cornu ammonis 1 (CA1) area; probably through reduction of astrocytes and microglia activation in the ischemic CA1 area, thus attenuating neuroinflammation. Additionally, it exerts antioxidant effects by increasing the SOD1 and SOD2 expression in the CA1 area [239]. Another study reported similar results against transient global cerebral ischemia in high-fat diet gerbils. A commercially available fucoidan extracted from *F. vesiculosus*, at 50 mg/kg daily, efficiently reduced oxidative stress, in pre- and post-ischemic phases, by increasing the expression of the antioxidant enzymes, namely SOD1 and SOD2, thus preventing neuronal cell death in CA1–3 regions [240]. At 100 μg/mL, the same fucoidan combined with non-invasive low intensity pulsed electric field (LIPEF) at 60 V/cm had neuroprotective activity in mouse motor neuron-like cell line NSC-34 against H_2_O_2_-induced oxidative and endoplasmic reticulum (ER) stress. These combination treatments attenuated GSH depletion in the H_2_O_2_-treated NSC-34 cells, controlling the ratio of GSH/GSSG. The same study reported that BiP expression is increased by both single treatments, while this effect is enhanced with the combination of treatments. BiP is an essential Ca^2+^-binding protein maintaining ER homeostasis [241].

**Figure 2:**
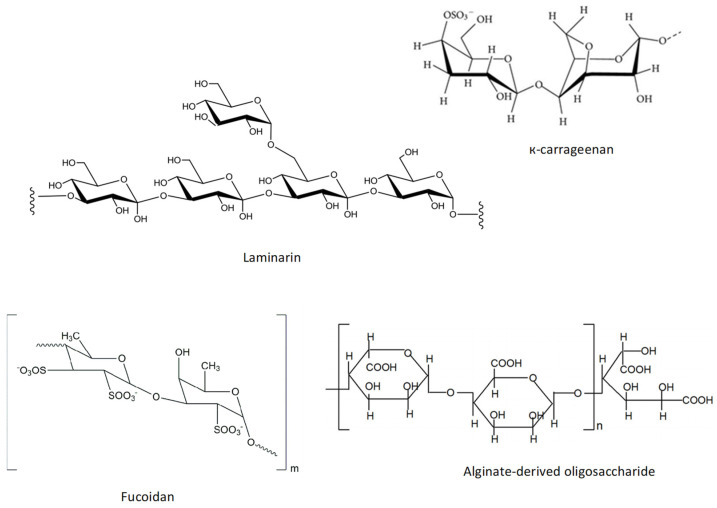
Chemical structure of к-carrageenan and laminarin, and schematic representation of the chemical structure of fucoidan and alginate-derived oligosaccharide.

Fucoidans extracted from *Sargassum hemiphyllum* (Turner) C. Agardh may be enhanced by compressional-puffing pretreatment, demonstrated in SH-SY5Y cells treated with 6-OHDA. The compressional-puffing pretreatment increased the extraction yield and molar ratios of sulfate/fucose of fucoidan and decreased molecular weight and impurities of fucoidan. All fucoidan extracts obtained from changing puffing (0 kg/cm^2^, 1.7 kg/cm^2^, and 10.0 kg/cm^2^) revealed antioxidant activity. The antioxidant activity against DPPH^●^ revealed IC_50_ values ranging from 1.72 to 2.58 mg/mL and in 2,2-azinobis(3-ethylbenzothiazoline-6-sulfonate radical cation (ABTS^●+^) assay IC_50_ ranging from 0.17 to 0.34 mg/mL. The extract obtained applying 10.0 kg/cm^2^ puffing revealed the most effective neuroprotection effects, presenting a high molar ratio of sulfate/fucose (1.74) [242] (Table 2). The general structure of fucoidan is presented in Figure 2.

Porphyran is a sulfated galactan found in the cell wall of *Porphyra* spp. [243]. Oligo-porphyran (prepared by acid hydrolysis of porphyrin and mainly composed of sulfated galactans and oligosaccharides with a linear backbone of alternating 3-linked β-D-galactose and 4-linked α-L-galactose-6-sulfate) from *Porphyra capensis* Kützing, at 50 mg/kg, showed neuroprotective effects by regulating the phosphatidylinositol-3 kinase/protein kinase-B/B cell lymphoma-2 (PI3K/Akt/Bcl-2) signalling pathway, thus contributing to counteracting the neurobehaviour deficits in an animal model of PD induced by MPP^+^ [244] (Table 2). Activation of the PI3K/Akt/Bcl-2 signal pathway leads to neuronal survival, with Bcl-2 being an anti-apoptotic protein [245].

**Table 2:** Selected studies on the neuroprotective effect of polysaccharides present in macroalgae.

Compound	Concentration Tested	Macroalgae	Bioactivity	Type of Model	Experimental Model	Reference
Ulvan	20–333.33 µg/mL	*U. lactuca*	Antioxidant	In vitro	ABTS^•+^, DPPH^•^, ^•^OH scavenging, and metal chelating activity	[203]
	33–133 µg/mL	*U. lactuca*	Inhibition AChE and BChE	In vitro	In vitro assay	[203]
Fucoidan	1–50 µg/mL	*F. vesiculosus*	Mitochondria biogenesis/energy production	In vitro	PD-induced by MPP^+^ in SH-SY5Y (parameters assessed: cell viability, apoptosis, oxidative stress, and mitochondrial dysfunction)	[232]
Fucoidan	25–100 µg/mL	*F. vesiculosus*	Anti-inflammatory	In vitro	LPS -induced inflammation in BV2 microglia cells (parameters assessed: cell viability, levels of ^•^NO and PGE_2_, and expression of pro- and anti-inflammatory mediators)	[234]
Fucoidan	3.125–100 µg/mL	*F. vesiculosus*	Anti-aggregation Aβ_1–42_	In vitro	H_2_O_2_ or Aβ_1–42_ treated PC-12 cells (parameters assessed: cell viability, apoptosis, and neurite outgrowth)	[236]
Fucoidan	3.125–100 µg/mL	*U. pinnatifida*	Anti-aggregation Aβ_1–42_	In vitro	H_2_O_2_ or Aβ_1–42_ treated PC-12 cells (parameters assessed: cell viability, apoptosis, and neurite outgrowth)	[236]
Fucoidan		*S. hemiphyllum*	Antioxidant	In vitro	ABTS^•+^, DPPH^•^, FRAP	[242]
Porphyran	25 and 50 mg/kg	*P. capensis*	Anti-apoptotic	In vivo	Male C57BL6 mice model of PD induced by MPTP (parameters assessed: body weight ratio and behavioural patterns)	[244]
Sulphated agaran	15–60 µg	*Gracilaria cornea*	Antioxidant	In vivo	PD rat model induced by 6-OHDA (parameters assessed: behavioural, neurochemical, and transcriptional analyses)	[246]
K-carrageenan	0.01–1.0 mg/mL	*H. musciformis*	Anti-apoptotic	In vitro	SH-SY5Y cells treated by 6-OHDA (parameters assessed: cell viability, apoptosis, mitochondrial potential, and H_2_O_2_ levels)	[247]

Carrageenans are polysaccharides found in Rhodophyta with an enantiomeric variation, D- or L-, in 4-linked α-galactose. Kappa-carrageenan extracted from *Hypnea musciformis* (Wulfen) J.V. Lamouroux exert neuroprotection against neurotoxicity induced by 6-OHDA on SH-SY5Y cells at concentrations of 0.6 and 1 mg/mL. This compound reduces the loss of mitochondria transmembrane potential and reduces the caspase-3 activity, improving cell viability [247] (Table 2). Kappa-carrageenan structure is shown in Figure 2.

Ulvans, present in Chlorophyta species, are polyanionic heteropolysaccharides from cell walls composed predominantly of rhamnose, glucuronic acid, iduronic acid, and xylose. Other monosaccharides are often present, such as glucose, galactose, arabinose, and mannose [248]. SPs extracted from *U. lactuca* showed antioxidant activity by scavenging ABTS^●+^ and DPPH^●^ at the concentration range of 83.33–333.33 μg/mL, while scavenged ^●^OH radicals at 25–100 μg/mL. In addition, they exhibited an inhibitory effect on AChE and BChE, with an IC_50_ of 106.93 µg/mL and 93.45 µg/mL, respectively [203] (Table 1).

Laminarin (Figure 2) is a polysaccharide composed of (1,3)-β-D-glucan with β(1,6) branching, particularly abundant in *Laminaria* spp. [249]. This polysaccharide at 50 mg/kg demonstrated that it can attenuate oxidative stress and neuroinflammation by increasing the expression of SOD and anti-inflammatory cytokines such as IL-4 and IL-13 in CA1 pyramidal neurons in gerbils before and after ischemia/reperfusion injury [250]. Another study demonstrated that the administration of 50 mg/kg laminarin as pre-treatment of transient forebrain ischemia in gerbils effectively reduced microglial activation [251].

The alginate-derived oligosaccharide prepared by oxidative degradation from alginate (average molecular weight is 1500 Da) suppressed microglial activation in LPS/Aβ-induced neuroinflammation in BV2 cells. Pretreatment of BV2 microglia with alginate-derived oligosaccharide prior to LPS/Aβ stimulation led to significant inhibition of the production of ^●^NO and prostaglandin E2 (PGE2), expression of iNOS and COX-2, and secretion of proinflammatory mediators. These effects resulted from the attenuation of TLR4 and NF-κB overexpression [252]. TLR4 activates NF-κB and regulates pro-inflammatory responses [253]. In addition, the authors of this study reported that the alginate-derived oligosaccharide promoted phagocytosis of Aβ_1–42_ aggregates by its interaction with TLR4 [252]. The schematic representation of the chemical structure of this polysaccharide is presented in Figure 2.

A seleno-polymannuronate (Se-PM, at 0.5 mg/mL) prepared from alginate-derived polymannuronate (PM) in comparison with sulfated PM (S-PM) and PM, had better neuroprotection activity by inhibiting the aggregation of Aβ_1–42_ and reducing BACE1 and cytochrome c expression in N2a-SW cells (murine neuroblastoma N2a cell stably transfected with human mutant APP695). Besides that, it normalized the ratio of Bax and Bcl-2 and enhanced the mitochondrial membrane potential in N2a-SW cells [254]. BACE1, also known as β-secretase, cleaves APP generating Aβ, which may aggregate, compromising cognitive and motor functions. [255].

Sulfated agaran isolated from *Gracilaria cornea* J. Agardh through protease digestion by papain, at a single intrastriatal injection administration of 60 μg, increased BDNF transcription, improving behaviour in a rat model of PD induced by 6-OHDA. The same study reported antioxidant and anti-inflammatory effects in vivo. Sulfated agaran at 15, 30, and 60 μg, intrastriatal administrated, showed reduced NO_2_ and NO_3_ levels, while the same compound at 30 and 60 μg increased GSH levels. Sulfated agaran at 60 μg also reverted the 6-OHDA-induced increase of inflammatory factors such as IL-1β, and iNOS, in the striatum. These effects occurred possibly via NF-κB inhibition [246] (Table 2).

Fucoidan, laminarin, and alginate extracted from *Sargassum polycystum* C. Agardh, *Turbinaria ornata* (Turner) J. Agardh and *Padina boryana* Thivy were tested as scavengers of DPPH^●^, O_2_^●−^, and ^●^OH. All compounds (stock solution of 2 mg/mL) displayed DPPH^●^ scavenging activity in the range of 60–80% and ^•^OH scavenging activity between 40 and 90%. These compounds were less active against superoxide anion radicals [256].

Sodium oligomannate is a mixture of oligosaccharides extracted from the seaweed *Ecklonia kurome* Okamura used in China as a treatment for mild to moderate AD and to improve cognitive function [257]. The mechanism of action of this compound is unclear but was reported to inhibit the toxicity induced by Aβ in both cortical cells and the SH-SY5Y cell line; inhibit the apoptosis induced by Aβ in SH-SY5Y by reducing the high concentration of intracellular Ca^2+^; and suppress the generation of ROS. Furthermore, sodium oligomannate blocked the Aβ fibril formation, which may be responsible for its anti-cytotoxic effects, inducing amyloid-beta disaggregation, regulating inflammatory responses to amyloid plaques, protein binding inside neurons, and modulating the gut microbiota [257,258].

### 3.2. Aminoacids, Peptides, and Protein Hydrolysates

Bioactive peptides are nitrogen and amino acid sources and possess properties such as immunomodulatory, antibacterial, antithrombotic, antihypertensive, and neuroprotective effects [259,260,261,262].

Proteinaceous factors such as neurotrophins, growth factors, neurotrophic cytokines, and neuroprotective peptides promote neuronal survival in physiological and pathological conditions [263]. Therefore, these proteinaceous factors can be helpful strategies against neurodegenerative disease.

The protein content of seaweed varies significantly among species. The red seaweed species have a higher protein content than other algae. Red macroalgae contain almost 47% (*w*/*w* dw), green seaweeds contain 9–26% (*w*/*w* dw), while brown algae contain 3–15% (*w*/*w* dw) [17]. In addition, red seaweeds have the highest ratio between essential and non-essential amino acids, which is in the range of 0.98–10.2 [264].

Most seaweeds are a good source of essential amino acids and contain bioactive amino acids and peptides such as taurine, carnosine, and GSH. The content of both bioactive amino acids, peptides, and essential amino acids varies significantly among species [265,266].

**Figure 3:**
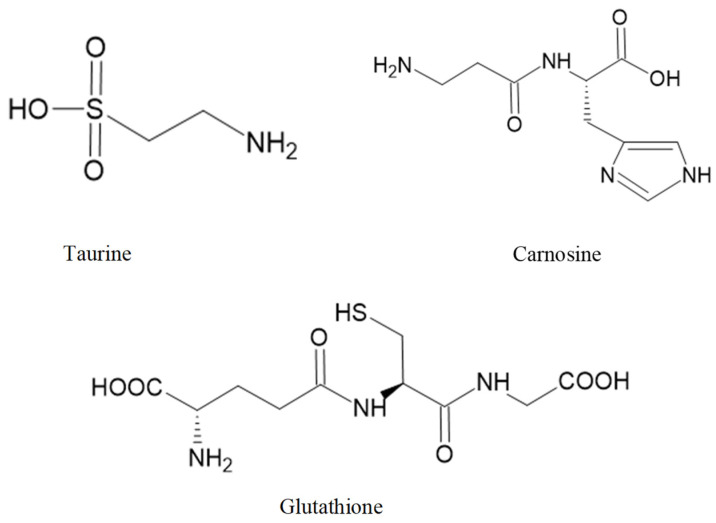
Some bioactive amino acids and peptides with neuroprotective activities.

Taurine (Figure 3) is biosynthesized from two amino acids, cysteine and methionine, and both are sulfur-containing amino acids. This amino acid is crucial for numerous biological and physiological functions, including bile salts formation, retinal and neurological development, osmoregulation, modulation of cellular Ca^2+^ level, and immune function [267,268].

Taurine levels are higher in Rhodophyta species compared to other classes. In *Saccharina latissima* (L.) C.E. Lane, C. Mayes, Druehl, et G. W. Saunders and *Porphyra tenera* Kjellman taurine levels are around 400 mg/g dry weight [269]. In a mouse model of PD induced by paraquat (1,1′-dimethyl-4-4′-bipyridinium dichloride) and maneb, taurine (at 150 mg/kg) administrated for six consecutive weeks (twice per week) showed neuroprotection activity in dopaminergic and noradrenergic neurons by inhibition of microglial M1 polarization [270,271]. Microglial M1 polarization has pro-inflammatory effects and can be activated by protein aggregates such as α-synuclein, Aβ, and tau oligomers [272].

In a recent study by Terriente-Palacios et al. [273], amino acids and sulfonic acid derivatives such as taurine, its precursor hypotaurine, and the homologue homotaurine, were quantified in 26 different species of commercial macroalgae, microalgae, and algae-enriched food products from the market. These sulfonic acid derivatives are bioactive molecules which may provide protection against free radicals and heavy metals and modulate several diseases [273]. These authors reported that taurine and its analogues were presented in higher quantities in red species, followed by green and brown species. High quantities of homotaurine were found in green algae *U. lactuca* and *Gracilaria vermiculophyla* (Ohmi) Papenfuss, as well as in the brown algae *U. pinnatifida* [273]. Homotaurine (also known as tramiprosate) is an orally administered compound that binds to Lys16, Lys28, and Asp23 of Aβ42, stabilizing its monomers, thus reducing oligomeric and fibrillar (plaque) amyloid aggregation. The inhibition of oligomer formation and elongation provides neuroprotection against Aβ-induced subsequent deposition [274]. Besides the effects on amyloid aggregation, this compound also shows anti-inflammatory effects, and its molecular structure is related to the neurotransmitter GABA, acting as a functional agonist [274]. In AD patients, homotaurine reduced global cognitive decline in APOE4 allele carriers, indicating a disease-modifying effect [275].

Carnosine (Figure 3) can be isolated from the red seaweed *Ancanthophora delilei* J.V. Lamouroux [259]. Carnosine is an endogenous dipeptide (β-alanyl-L-histidine) abundantly distributed in the nervous tissues of several animal species [276]. Carnosine can regulate extracellular glutamate levels and prevent neuronal cell death [277]. Furthermore, in a rat model of intracerebral haemorrhage, carnosine can reduce inflammation by inhibiting the microglia activation and attenuating the oxidative stress by increasing GPx and SOD activities [278].

GSH (Figure 3) is found in *Ulva* spp. [279]. GSH, a tripeptide (γ-glutamylcysteinyl glycine), is a thiol-containing molecule and plays an important role in maintaining redox homeostasis [280]. Sulfhydryl residues in the thiol group of GSH molecules are easily oxidized into GSH disulfide (GSSG). Failure in GSH metabolism and GSH depletion is involved in the pathogenesis of ageing-related disease [281]. GSH is present in mitochondria, ER, and the nucleus, generally in reduced form. The oxidized form (GSSH) is produced during redox reactions with GSH consumption. Oxidative stress reduces the ratio of GSH/GSSG [282]. Depletion of intracellular GSH promotes mitochondrial ROS production and triggers mitochondrial membrane depolarization [283].

The chemical structure of these bioactive compounds is presented in Figure 3.

Harnedy et al. [284] found a novel decapeptide (Ser-Asp-Ile-Thr-Arg-Pro-Gly-Gly-Gln-Met) with antioxidant activity from an aqueous extract of *Palmaria palmata* (L.) F. Weber & D. Mohr. The oxygen radical absorbance capacity (ORAC) and ferric-reducing antioxidant power (FRAP) activity of this decapeptide showed values of 152.43 ± 2.73 and 21.23 ± 0.90 nmol Trolox equivalents (TE)/μmol peptide, respectively [284] (Table 3).

A novel peptide (Glu-Leu-Trp-Lys-Thr-Phe) isolated from *Gracilariopsis lemaneiformis* (Bory) E. Y. Dawson, Acleto, & Foldvik proteins through the hydrolysis mediated by different proteases showed antioxidant activity against DPPH^●^ with an EC_50_ of 1.514 mg/mL [285] (Table 3). Another novel peptide (KAQAD) isolated from *Pyropia yezoensis* (Ueda) M.S. Hwang & H.G. Choi showed anti-inflammatory effects in the mouse macrophage cell line RAW 264.7. The authors of this study reported inhibition of 66.67% in ^●^NO production at 1.000 ng/mL of the peptide. The anti-inflammatory activity of this bioactive peptide is related to the downregulation of ERK, protein 38, and JNK phosphorylation [286] (Table 3).

Through in silico studies, bioactive peptides were identified in *Caulerpa taxifolia* (M. Vahl) C. Agardh within the ribulose-1,5-bisphosphate carboxylase/oxygenase (RuBisCO) sequence. These peptides, whose ID numbers are H9EHL5 and H9EHK8, showed the capacity to activate ubiquitin-mediated proteolysis [287] (Table 3).

A glycoprotein isolated from *L. japonica* at a 100 μg/mL concentration showed anti-inflammatory activity by inhibiting LPS-induced pro-inflammatory mediators in BV2 microglial cells. These pro-inflammatory mediators included ^●^NO, PGE2, iNOS, and pro-inflammatory cytokines (IL-1β and TNF-α) [288]. Another glycoprotein isolated from *U. pinnatifida* showed inhibitory activities of BACE1 with an IC_50_ of 73.35 μg/mL, inhibition of AChE and BChE with an IC_50_ of 63.56 μg/mL and 99.03 μg/mL, respectively [204] (Table 3).

**Table 3:** Selected studies on the neuroprotective effect of protein hydrolysates from macroalgae.

Compound	Concentration Tested	Macroalgae	Bioactivity	Type of Model	Experimental Model	Reference
Glycoprotein (UPGP, 10 KDa)	5–100 µg/mL (cell viability) 50–500 µg/mL (COX inhibition) 0–5 mg/mL (SOD and xanthine oxidase inhibition)	*U. pinnatifida*	Inhibition of BACE1, AChE, and BChE and anti-inflammatory activity	In vitro	Inhibition of BACE1, AChE, BChE, COX-1, COX-2, SOD, and xanthine oxidase; assessment of ^•^NO level and cell viability of LPS-treated RAW 264.7 cells; determination of cell viability of primary hippocampal neurons	[204]
Ser-Asp-Ile-Thr-Arg-Pro-Gly-Gly-Gln-Met		*P. palmata*	Antioxidant	In vitro	ORAC and FRAP	[284]
Glu-Leu-Trp-Lys-Thr-Phe (ELWKTF)	2 and 4 mg/mL	*G. lemaneiformis*	Antioxidant	In vitro	DPPH^•^ scavenging activity	[285]
KAQAD	250–1000 ng/mL	*P. yezoensis*	Anti-inflammatory	In vitro	RAW 264.7 treated by LPS (parameters assessed: cell viability, levels of ^•^NO and ROS, and expression of pro- and anti-inflammatory mediators)	[286]
H9EHL5		*C. taxifolia*	Proteolysis activator	In silico	In silico assays	[287]
Glycoprotein (LJGP)	25–100 µg/mL	*L. japonica*	Anti-inflammatory	In vitro	LPS-induced proinflammation in BV2 microglial cells (parameters assessed: cell viability, levels of ^•^NO, PGE_2_, TNF-α, and IL-1β, expression of pro- and anti-inflammatory mediators, and activation of anti-inflammatory pathways)	[288]

### 3.3. Polyunsaturated Fatty Acids

Polyunsaturated fatty acids (PUFAs) can be divided into two families: ω-6 and ω-3. ω-6 is biosynthesized from linoleic acid (LA), and ω-3 is biosynthesized from α-linolenic acid (ALA). α-linolenic acid (α-ALA, C18:3, ω-3), eicosapentaenoic acid (EPA, C20:5, ω-3), docosahexaenoic acid (DHA, C22:6, ω-3), stearidonic acid (SDA; 18:4 ω-3), and docosapentaenoic acid (DPA, C 22:5, ω-3), are included in the ω-3 family; while γ-linoleic acid (γ-LA, C18:3, ω-6) and arachidonic acid (AA, C20:4, ω-6) belong to the ω-6 family [289,290].

PUFAs are essential fatty acids because mammals cannot synthesize them [291]. They contribute to several brain functions, including membrane fluidity, the function of ion channels, neurotransmitter production and activity, and signal transduction, controlling the activity of neurotransmitters and neuronal growth factors [289].

The possible mechanisms for neuroprotection of longer chain ω-3 fatty acids supplementation have been related to modulating the neuronal membrane, neurotransmission, signal transduction, and neural plasticity [292]. Furthermore, variations in the content of brain PUFAs are influenced by age, with longer chain (LC)-PUFA levels decreasing with ageing [291].

Macroalgae are a good marine source of ω-3 long-chain PUFAs, and their content varies among species and can suffer seasonal variations. ω-3 and ω-6 PUFA concentrations can range from 2 to 14 mg/g dry matter. It has been reported that Chlorophyta contains high concentrations of C16 and C18 PUFAs such as LA, C18:3, ω-6, α-ALA, C18:3, ω-3, and the Rhodophyta species such as *P. palmata* and *Porphyra* sp. are rich in EPA. The concentration of EPA in red algae can comprise 36% of total fatty acids [293,294,295].

Neuroinflammation is a crucial target in neurodegenerative diseases, and it has been reported that ω-3 PUFAs can ameliorate inflammation by reducing the expression of pro-inflammatory factors, including IL-6, IL-1β, and TNF-α [296]. Oxylipins are derived from PUFAs and can play a role in inflammation: the oxylipins derived from ω-3 PUFAs usually exert anti-inflammatory activity, while those generated from ω-6 PUFAs are generally present in pro-inflammatory activity. For example, PGE2 is a pro-inflammatory oxylipin generated from AA [297]. Furthermore, the imbalance between ω-6 and ω-3 PUFAs is known to cause inflammatory processes in the body [298].

Dong et al. [299] reported that EPA could normalize the relationship between ω-3 and ω-6 PUFAs in the rat hippocampus. Additionally, it was demonstrated that supplementation with EPA diet (0.8% ethyl-EPA) for 42 days in rats injected intracerebroventricular (from day 36 to 42) with the pro-inflammatory cytokine IL-1β could prevent the down-regulation of the expression of BDNF and its TrKB receptor [299].

DHA-rich algae oil, with EPA at 317 mg/g and DHA at 556 mg/g, can inhibit ERK expression to reduce levels of pro-inflammatory mediators-iNOS, IL-1β, and TNF-α in rats’ daily gavage with this algae oil after previous ischemic optic neuropathy induction for seven days. Additionally, it increased the expression level of a ciliary neurotrophic factor [300].

Clementi et al. [301] reported that pre-treatment of DHA, at 60 µM, can protect rat PC12 cells against H_2_O_2_-induced oxidative damage by activating the nuclear factor erythroid-derived 2-like 2/heme-oxygenase-1 (NFE2L2/HO-1) signalling pathway. In addition, it has anti-apoptotic effects by inhibiting Bax and activating Bcl-2 expression [301]. NFE2L2, an antioxidant transcription factor, encodes Nrf2, and the HO-1 is the primary target gene. When this pathway is activated, it promotes antioxidant, anti-inflammatory, and anti-apoptotic effects contributing to reducing high levels of intracellular ROS and increasing the intracellular levels of enzymatic antioxidants such SOD, CAT, and GPx [302,303].

**Figure 4:**
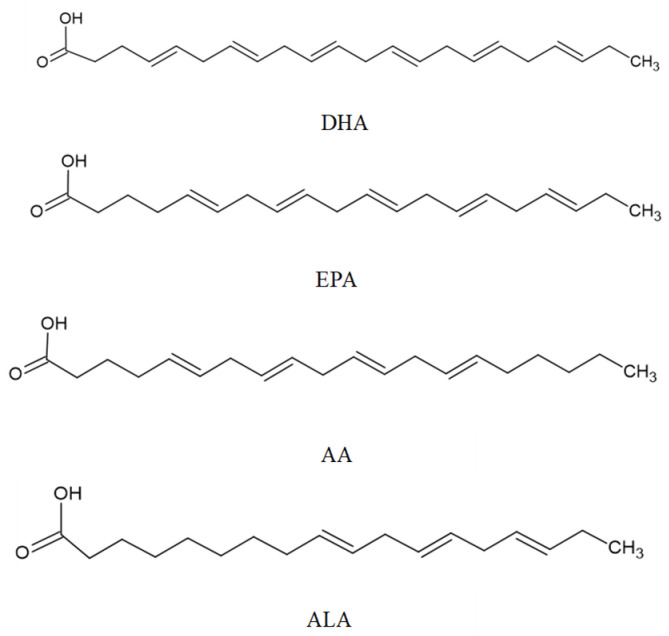
Main PUFAs with neuroprotection activities.

In a recent study, Souza et al. [304] demonstrated that the ingestion of a diet supplemented with 39% EPA and 24% DHA revealed neuroprotective effects against Paraquat (0.8 mM) -induced neuronal and mitochondrial impairments in *Drosophila melanogaster*. These neuroprotective effects reduced oxidative stress and mitochondria membrane permeability [304]. ω-3 PUFAs (51.8% EPA and 21.2% DHA) supplementation in 19-month-old mice proved increased hippocampal neurogenesis and dendritic arborization of newborn neurons. The improved hippocampal cognitive functions are related to enhanced cellular plasticity. After four weeks of dietary supplementation, improved object recognition and spatial and localization memory were observed [305]. The chemical structure of these ω-3 PUFAs is presented in Figure 4.

Mohibbullah et al. [306] pointed out that the high content of AA (7.5 μM) in ethanolic extract of *Gracilariopsis chorda* (Holmes) Ohmi is responsible for neuroprotective effects against oxidative stress in the hypoxia/reoxygenation model of rat hippocampal neurons. However, concentrations greater than 7.5 μM may cause cell death [306]. AA is present in the membranes of neuronal cells and can act as a second messenger involved in the regulation of signalling enzymes. Furthermore, the breakdown of AA by cyclooxygenases produces prostaglandins involved in inflammatory processes [307].

Fang et al. [207] reported that α-linolenic acid extracted from *Gloiopeltis furcata* J. Agardh inhibits AChE and BChE with an IC_50_ of 12.50 and 15.89 μg/mL, respectively.

### 3.4. Photosynthetic Pigments

Chlorophylls, phycobiliproteins, and carotenoids are the basic pigments found in seaweed that have a crucial role in photosynthesis [308].

**Figure 5:**
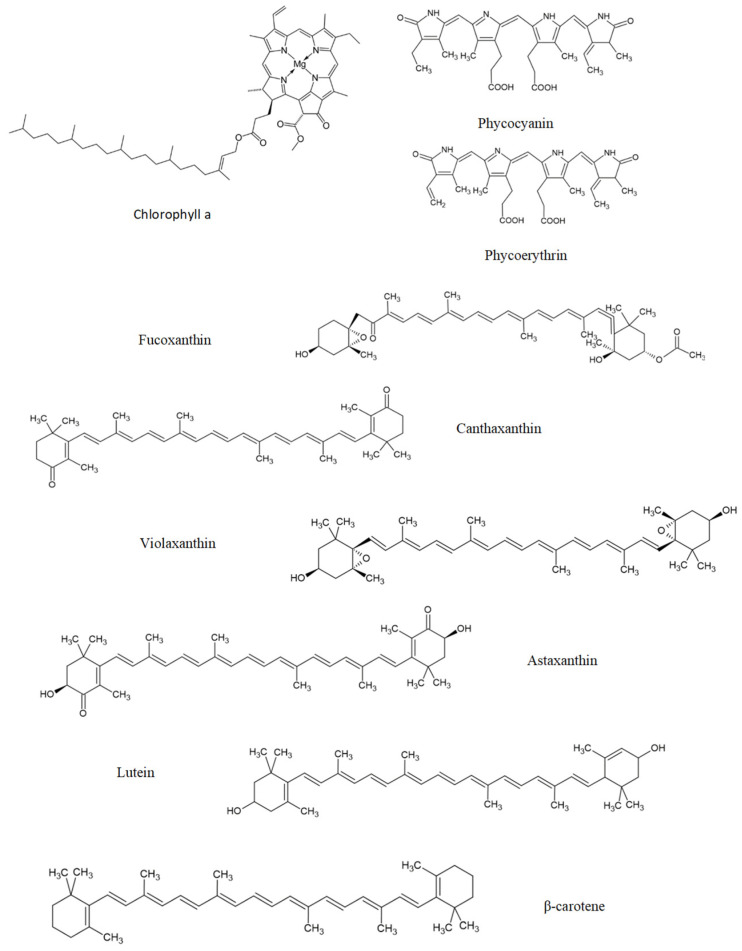
Some photosynthetic pigments with neuroprotection activities, namely, chlorophylls (chlorophyll a), phycobiliproteins (phycocyanin and phycoerythrin), and carotenoids (fucoxanthin, canthaxanthin, violaxanthin, astaxanthin, lutein, and β-carotene).

Chlorophyll a (Figure 5) and pheophytin a (derived from chlorophylls) at 5 μg/mL and 2.5 μg/mL, respectively, can decrease pro-inflammatory cytokines and chemokine levels in BV2 cells stimulated with LPS and interferon-gamma (IFN-γ). In addition, they suppress NF-κB activation and signalling mediators such as STAT-1 and interferon regulatory factor (IRF)-1 [250].

Phycobiliproteins are a group of hydrophilic accessory pigments that comprises red-coloured phycoerythrins and blue-coloured phycocyanins. Light is captured by phycoerythrin during photosynthesis, transferred to phycocyanin and then through allophycocyanin to the central chlorophyll, a molecule in the photosystem complex [309]. The structure of phycobiliproteins is presented in Figure 5.

Phycobiliproteins (phycobilin at 100 or 500 μg/mL) and chlorophyll a at 245 μg/mL from *P. palmata* revealed anti-inflammatory properties by decreasing inflammatory mediators, namely IL-6, TNF-α, and ^●^NO, in LPS-stimulated murine macrophages (RAW 264.7 cells) [310] (Table 4 [310,311,312,313,314,315,316]). Phycocyanin (48 mg/mL) stimulated the oxidative stress response in a yeast model of PD (alpha-synuclein induced toxicity) by modulating transcript levels of genes related to oxidative stress, such as SOD2 and HAP4 [311].

A docking study reported that β-carotene (carotenoid), phycoerythrin, and phycocyanin can act as antagonists of EphA4 and histone deacetylase, as a good strategy for treating AD and ALS. These pigments interact with EphA4 and histone deacetylase by van der Waals interaction and, especially, hydrogen bonds [317]. Inhibition of the axon guidance protein EphA4 revealed axonal regeneration, promoting axon growth [318]. High histone deacetylase activity occurs in neurodegenerative diseases, their inhibition stimulating neurogenesis and synaptic plasticity, and still, inhibition of histone deacetylase can regulate several pathways involved in neuroinflammation and apoptosis [319].

Carotenoids are a class of pigments widely distributed in nature [320]. There are more than 750 carotenoids in nature, of which 250 are of marine origin [321]. Carotenoids are isoprenoid molecules synthesized de novo by photosynthetic plants, fungi, and algae [322]. These compounds are responsible for the yellow, orange, and red colouration of plants and algae [323]. In other organisms, carotenoids are secondary metabolites generated by enzymatic reactions [324].

Carotenoids can be classified into two groups, carotenes and xanthophylls. Carotenes such as α-carotene, β-carotene, and lycopene are hydrocarbons lacking oxygen and xanthophylls contain oxygen, such as lutein, astaxanthin, zeaxanthin, and β-cryptoxanthin [325,326]. Both classes of carotenoids can have acyclic or cyclic compounds [327].

Carotenoids can act as antioxidants during photosynthesis, protecting the photosynthetic apparatus from oxidative damage [320]. In many other species, some carotenoids are precursors of vitamin A [328]. Humans cannot synthesize carotenoids, obtaining them from the diet [329]. These compounds have several therapeutic properties, including antioxidant [330], anticancer [331], and prevention and potentially the management of neurodegenerative disorders [332].

Chemically, carotenoids are C40 hydrocarbons with isoprenoids as building units [332]. Most carotenoids have high lipophilicity and, therefore, can cross the blood–brain barrier (BBB) [333]. The human brain contains various carotenoids, including α-carotene, α-cryptoxanthin, β-carotene, β-cryptoxanthin, lutein, lycopene, and zeaxanthin. These carotenoids provide neuroprotection against oxidative stress [332]. The antioxidant activity is related to structural features of pigments such as the porphyrin ring, phythyl chain, and extended systems of conjugated double bonds [334].

The serum levels of some carotenoids, such as α- and β-carotenes, are lower in PD patients because these levels in serum positively correlate with levels in the brain [335]. On the other hand, the high serum levels of lycopene, lutein, and zeaxanthin were associated with a lower risk of AD mortality in aged people [336].

Fucoxanthin is one of the most abundant marine carotenoids corresponding to about 10% of total natural carotenoid production [337]. Fucoxanthin has been isolated from marine brown seaweeds, such as *Eisenia bicyclis* (Kjellman) Setchell, *U. pinnatifida*, *F. vesiculosus*, *L. japonica*, and others [338]. In addition, fucoxanthin is one carotenoid with an allenic bond that contributes to high antioxidant activity [339]. Therefore, fucoxanthin and its beneficial effects on neurodegenerative diseases have been studied extensively.

During the metabolism of fucoxanthin, it is deacetylated by lipase and esterase from the pancreas or in intestinal cells into fucoxanthinol [338]. Fucoxanthin at 0.075 μg/mL and this derivative, at the same concentration, showed antioxidant effects in primary cultures of rat hippocampal neurons against hypoxia-induced oxidative stress [312] (Table 4).

Fucoxanthin intracerebroventricular injection at 0.05 mmol/L attenuated oxidative stress and apoptosis by activating the Nrf2-ARE and Nrf2-autophagy pathways and stimulating the action of antioxidant enzymes such as CAT and SOD in a mice model of traumatic brain injury [340]. Furthermore, this marine carotenoid, at 5 μM, 10 μM, and 20 μM, can activate the Nrf2/HO-1 signal pathway in a rat model of cerebral ischemic/reperfusion injury [341]. As mentioned before, the activation of this pathway is related to antioxidant, anti-inflammatory, and anti-apoptotic effects. In LPS (100 ng/mL)-activated BV-2 cells, one more time, fucoxanthin at 20 μM significantly activated Nrf-2/HO-1 and PKA/CREB pathways, therefore, suppressing the expression of ^●^NO and PGE2 by down-regulating iNOS and COX-2 [342]. Similar to Nrf-2, PKA/CREB pathways are involved in mitochondrial biogenesis [343].

**Table 4:** Selected studies on the neuroprotective effect of pigments from macroalgae.

Compound	Concentration Tested	Macroalgae	Bioactivity	Type of Model	Experimental Model	Reference
Fucoxanthin	50–200 mg/kg	*S. horneri*	Inhibition of AChE and animal behaviour	In vitro and in vivo	In vitro AChE inhibition and scopolamine-induced cognitive impairment in ICR mice (parameters assessed: locomotor activity, recognition impairment, spatial learning and memory impairments, expression of neurotropic factors, and ChAT and AChE activity)	[205]
Fucoxanthin	2–100 µM	*U. pinnatifida*	Inhibition of BACE1	In vitro	In vitro assay	[208]
Fucoxanthin	2–100 µM	*E. stolonifera*	Inhibition of BACE1	In vitro	In vitro assay	[208]
Fucoxanthin	100–400 µM	*E. bicyclis*	Inhibition of hMAOs	In vitro	In vitro assay	[211]
Fucoxanthin	100–400 µM	*U. pinnatifida*	Inhibition of hMAOs	In vitro	In vitro assay	[211]
Extract and fractions containing phycobiliproteins and chlorophyll a	100–500 µg/mL	*P. palmata*	Anti-inflammatory	In vitro	LPS-stimulated RAW 264.7 cells (parameters assessed: Levels of ^•^NO, TNF-α, and IL-6)	[310]
Ethanol extract, fucoxanthin, and fucoxanthinol	Extract (5–30 µg/mL) Fucoxanthin (25–250 ng/mL) Fucoxanthinol (50–100 ng/mL)	*U. pinnatifida*	Antioxidant	In vitro	Primary cultures of rat hippocampal neurons (parameters assessed: cell viability, apoptosis, mitochondrial integrity, intracellular ROS, and total length of primary neurites)	[312]
Fucoxanthin	0.1–30 µM (in vitro) 50–200 mg/kg (in vivo)	*S. horneri*	Inhibition of Aβ assembly	In vitro and in vivo	In vitro inhibition of Aβ_1–42_ oligomers formation; SH-SY5Y cells treated with Aβ_1–42_ oligomers (parameters assessed: cell survival); Aβ_1–42_ oligomer-treated mice (parameters assessed: locomotor activity, and recognition performance)	[313]
Ethyl acetate fraction containing fucoxanthin, canthaxanthin, and violaxanthin, among other compounds	10–100 µg/mL	*E. prolifera*	Antioxidant and anti-apoptotic	In vitro	HT-22 cells treated with glutamate (parameters assessed: cell viability, intracellular ROS, apoptosis, expression of antioxidant activities, and neurotropic factors)	[314]
Fucoxanthin	0.3–3 µM	*S. horneri*	Antioxidant and Inhibition of Aβ assembly	In vitro	SH-SY5Y cells treated with Aβ oligomers (parameters assessed: cell viability, apoptosis, intracellular ROS, and activation of signalling pathways)	[343]
Fucoxanthin	0.3–3 µM	*S. horneri*	Antioxidant	In vitro	H_2_O_2_-induced toxicity in SH-SY5Y cells and in primary cerebellar granule neurons (parameters assessed: cell viability, apoptosis, intracellular ROS, and signalling pathways activation)	[344]

It has been demonstrated that fucoxanthin, at 200 mg/kg, administrated before LPS treatment in mice, displayed anti-inflammatory action by suppressing the expression of AMPK-NF-κB and consequently inhibiting the release of neurotoxic mediators, such as iNOS and COX-2, and pro-inflammatory cytokines, such as TNF-α, IL-6, and IL-1β [344].

It has also been reported that fucoxanthin in the concentration range of 0.01–2 μM can ameliorate the Aβ aggregation and their related effects, such as oxidative stress and neuronal death. It was reported that this carotenoid reduces Aβ_1–42_ aggregation in PC-12 neuronal cells [345]. Xiang et al. [313] reported that fucoxanthin extracted from *Sargassum horneri* (Turner) C. Agardh, in the concentration range of 0.3–1 μM, could inhibit Aβ assembly in SH-SY5Y cells. Additionally, this inhibition was reinforced by hydrophobic interaction between this carotenoid and the Aβ peptide [313] (Table 4).

Fucoxanthin at 3.0 μM can attenuate Aβ oligomer-induced neurotoxicity and oxidative stress, possibly by activating the PI3K/Akt pathway and inhibiting the ERK pathway in the SH-SY5Y cell line [315] (Table 4). PI3K/AKT/mammalian target of the rapamycin (mTOR) is a survival pathway and promotes constitutive autophagy. While ERK signalling is related to proliferation and neuronal survival, it can also promote neural cell death and, thus, can be involved in the pathogenesis of neurodegeneration [245]. Furthermore, another study showed that fucoxanthin at 3 μM concentration activated the PI3K/Akt cascade and inhibited the ERK pathway in SH-SY5Y cells against H_2_O_2_-induced neurotoxicity [316] (Table 4).

In addition, this marine carotenoid can inhibit some enzymes. It was shown to inhibit BACE-1 in vitro, with an IC_50_ of 5.31 μM [208] (Table 4). Lin et al. [205] reported that fucoxanthin reduces AChE activity in the hippocampus and cortex of mice. Molecular docking studies showed that fucoxanthin inhibits AChE by a non-competitive mechanism because fucoxanthin can form hydrogen bonds with Asp283 and Ser286 residues in the peripheral anionic site (PAS) of AChE. An IC_50_ value of 81.2 µM was obtained experimentally in vitro.

According to the docking study conducted by Jung et al. [211], fucoxanthin showed higher binding affinity to human (h)MAOs, especially hMAO-B (−9.12 kcal/mol) than hMAO-A (−7.34 kcal/mol), with an IC_50_ of 211.12 μM and 197.41 μM, respectively. Additionally, fucoxanthin was also revealed to be a selective agonist of dopamine D3 and D4 receptors, involving interactions with residues of dopamine D3 (Val111, Thr115, and Ser196) and D4 receptors (Asp115 and Ser196), whose half-maximal effective concentration (EC_50_) was 16.87 and 81.87 μM, respectively [346].

Recently, it was reported that an *Enteromorpha prolifera* (O. F. Müller) J. Agardh extract rich in carotenoids, including fucoxanthin, canthaxanthin, and violaxanthin exerted antioxidant and anti-apoptotic actions in HT-22 cells treated with glutamate. At 100 µg/mL, the carotenoid-rich extract enhanced the expression of antioxidant enzymes via the activation of the TrkB/Akt pathway. HO-1, NAD(P)H quinone oxidoreductase-1 (NQO-1), and glutamate-cysteine ligase catalytic subunit (GCLC) are examples of these antioxidant enzymes [314] (Table 4).

Astaxanthin is a red fat-soluble pigment, and it is reported that it has more potent biological activity than other carotenoids and is used as a nutritional supplement in foods, nutraceuticals, and pharmaceuticals [347].

Trans-astaxanthin (at 80 mg/kg, for seven days) demonstrated preventive effects against LPS-induced pro-inflammatory cytokines such as IL-1β, IL-6, and TNF-α, as well as neurotoxic factors, such as iNOS, by regulating NF-κB in the hippocampus and prefrontal cortex in a mice model of neuroinflammation and depression [348]. Another study reported the anti-inflammatory effects of astaxanthin at 75 mg/kg in a rat model of subarachnoid haemorrhage [349].

It was also shown that astaxanthin attenuated glutamate-induced neurotoxicity in HT22 cells by regulating the Akt/GSK-3β signalling [350]. GSK3 regulates the balance of inhibitory/excitatory neurotransmission, regulating the function of various ionotropic neurotransmitter receptors, including GABA_A_, AMPA, and NMDA receptors. Akt is only the signal transduction mediator [351].

One study compared the neuroprotective activity of the astaxanthin, β-carotene, and canthaxanthin against Aβ 25–35 (0.01 μM) treated PC12 cells. Results demonstrated that astaxanthin at 0.5–10.0 μM provided adequate protection, while canthaxanthin provided moderate neuroprotective effects at 1.0–5.0 μM, and β-carotene revealed low protection activity at 10.0 μM [352].

In patients with AD, the supplementation with lutein, meso-zeaxanthin, and zeaxanthin at 10:10:2 mg/day plus 1 g/day of fish oil (430 mg DHA and 90 mg EPA), during 18 months revealed attenuation of the AD progression and improved memory, sight, and mood [353].

In a mouse model of ischemic stroke, lutein, at 0.2 mg/kg, regulated the NF-кB signalling pathway, decreased the expression of COX-2, and increased the expression of Bcl-2, thus attenuating neuroinflammation and apoptosis [354]. Another study reported the antioxidant effects of lutein at 15 and 30 mg/kg in a mice model of transient cerebral ischemia through regulation of the GSH/GSSG ratio and activation of antioxidant enzymes such as SOD, GPx, and catalase [355].

Hira et al. [335] reported that β-carotene, at 1.02 and 2.05 mg/kg, had potent antioxidant activity, increasing antioxidant enzymes (SOD and CAT) and GSH levels and decreasing the Aβ protein fragments in a mice model treated with streptozotocin (3 mg/kg, intracerebroventricular). Furthermore, it can bind to AChE, thus, inhibiting the enzyme and preventing cognitive decline [335]. Another study reported regulating the expression of Nrf2 and Keap1, reducing the oxidative stress induced by traumatic brain injury, in old male mice C57BL/6, treated with β-carotene at 30 mg/kg [356].

However, the potential pro-oxidant effects of carotenoids are also recognized. Carotenoids can react with ROS and RNS, leading to variations in the redox properties of carotenoids. For example, the formation of oxidation products from β-carotene comprises β-apo-carotenals and epoxy carotenoids, such as β-ionone, β-apo-14’-carotenal, β-apo-10’-carotenal, β-apo-8’-carotenal, and β-carotene 5,8-endoperoxide, resulting these products from the reaction of β-carotene with O_2_. These products are highly reactive [357].

The structure of the abovementioned pigments is presented in Figure 5.

### 3.5. Phlorotannins and Other Phenols

More than 150 phlorotannins are identified in brown macroalgae, these phenolic compounds having a significant structural heterogeneity. Phloroglucinol (1,3,5-trihydroxy benzene) by polymerization can lead to a large heterogeneity of these compounds [358]. According to the linkages between phloroglucinol units, phlorotannins can be classified into three primary types. The fucols are phlorotannins with only phenyl bonds, phlorethols with aryl ether linkages, and fucophlorethols with aryl ether and phenyl bonds [359].

**Figure 6:**
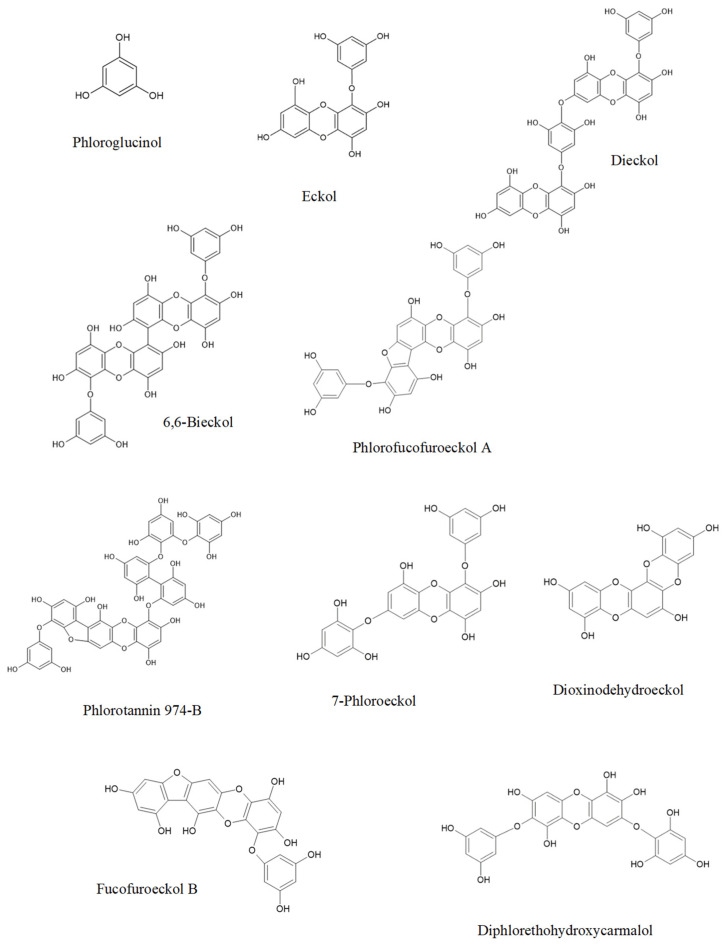
Phlorotannins that present neuroprotective activities.

Phlorotannins present in cell walls play essential roles as a defence mechanism against undesirable environmental conditions such as salinity level, nutrient and light availability, UV radiation, herbivores, and microbes [358,360]. The structure of phlorotannins is presented in Figure 6.

Antioxidant activity is the main bioactivity displayed by phenolic compounds, which can prevent cancer, neurodegenerative diseases, and other disorders [361].

*H. elongata* has demonstrated antioxidant activity against DPPH^●^, metal ions, lipid peroxides, and H_2_O_2_ due to high levels of phenolic compounds [362,363]. The 60% methanolic extract was characterized by a total phenolic content of 286.0 mg gallic acid equivalents/g, a total flavonoid content of 109.8 mg quercetin equivalents/g and condensed tannin content of 35.6 mg catechin equivalents/g [362].

Eckol and dieckol isolated from *E. bicyclis* showed reversible inhibitory effects on hMAO-A and hMAO-B. Eckol and dieckol inhibit hMAO-A/hMAO-B with IC_50_ values of 7.20 μM/83.44 μM and 11.43 μM/43.42 μM, respectively. The molecular docking analysis revealed that both phlorotannins exhibit higher binding affinity towards hMAOs through hydrogen bonding and hydrophobic interactions [212]. 6,6′-Bieckol (40 μM) extracted from *Ecklonia stolonifera* Okamura had anti-inflammatory effects against LPS (100 ng/mL)-stimulated BV2 and murine primary microglial cells. This phlorotannin can reduce the expression of iNOS and COX-2 in a dose-dependent manner and inhibit the activation of NF-κB, reducing the phosphorylation of JNKs, p38 MAPK, and Akt [364] (Table 5).

In addition, phlorofucofuroeckol-A also has noncompetitive inhibitory activity against both hMAO isoforms, showing higher selectivity for hMAO-B (IC_50_ = 4.89 µM) than for hMAO-A (IC_50_ = 9.22 µM). Besides that, phlorofucofuroeckol-A and dieckol were revealed to be dopamine receptor (D3R/D4R) agonists [213] (Table 5).

Several phlorotannins isolated from *E. bicyclis*, including dioxinodehydroeckol, eckol, and phlorofurofucoeckol A, showed non-competitive inhibition of BACE-1 with an IC_50_ of 5.35 μM, 12.20 μM, and 2.13 μM, respectively [209] (Table 5). The fucofuroeckol-b, whose molecular weight is 478.054 Da, inhibited BACE-1 with IC_50_ = 16.1 μM [210] (Table 5). The phlorotannin 974-B was isolated from the ethyl acetate (EtOAc) fraction obtained from the ethanolic extract of *E. bicyclis*. This phlorotannin demonstrated anticholinesterase activity against AChE and BChE with an IC_50_ of 1.95 and 3.26 μM, respectively [206]. The phlorotannin 974-A extracted from *E. stolonifera* competitively inhibited the mushroom tyrosinase activity towards the substrates l-tyrosine and l-DOPA with IC_50_ values of 1.57 and 3.56 µM, respectively. This inhibition is possible by forming hydrogen bonds between the hydroxyl residues of this compound and residues at the catalytic and allosteric sites of tyrosinase. Besides that, hydrophobic interactions stabilizing the protein-ligand interaction in the catalytic site occurred [365].

In addition, 7-phloroeckol (30 μM) isolated from EtOAc fraction obtained from *E. bicyclis* suggested potent neuroprotective effects against Aβ(25–35)-induced toxicity in PC12 cells through inhibition of ROS while maintaining Ca^2+^ homeostasis [366]. The molecular size and number of hydroxyl groups present in the phlorotannins are important factors responsible for their neuroprotective effects against Aβ-induced cytotoxicity.

Diphlorethohydroxycarmalol, at 50 μM, showed efficiently neuroprotective effects against oxidative stress induced by H_2_O_2_ (1.25 mM) in murine hippocampal neuronal cells (HT22). This phlorotannin decreased the intracellular Ca^2+^ level induced by oxidative stress and decreased the levels of pro-apoptotic proteins such as Bax, caspase-3, and caspase-9, increasing cell viability [367] (Table 5).

Phlorotannins with higher molecular weight have demonstrated better bioactivity. [366]. Bogolitsyn et al. [368] reported that the range of molecular masses of algal polyphenolic components for better antioxidant activity is 8–18 kDa. The increased value of this property can lead to mutual shielding of the reducing centres of the phlorotannins due to conformational changes of these molecules through the formation of intramolecular and intermolecular hydrogen bonds.

**Table 5:** Selected studies on the neuroprotective effect of phlorotannins from macroalgae.

Compound	Tested Concentration	Macroalgae	Bioactivity	Type of Model	Experimental Model	Reference
Dioxinodehydroeckol, eckol, phlorofurofucoeckol-A		*E. bicyclis*	Inhibition of BACE1	In vitro	In vitro assay	[209]
Fucofuroeckol-b	25–100 µg/mL	*E. bicyclis*	Inhibition of β-secretase	In vitro	Aβ-induced toxicity on SH-SY5Y cells overexpressing APP695swe (parameters assessed: cell viability and expression levels of sAPPβ and Aβ_42_)	[210]
**Eckol and dieckol**		*E. bicyclis*	Inhibition of hMAOs	In vitro	In vitro assay	[212]
Phlorofucofuroeckol-A		*E. stolonifera*	Inhibition of hMAOs	In vitro	In vitro assay	[213]
**6, 6′-Bieckol**	10–40 µM	*E. stolonifera*	Anti-inflammatory	In vitro	LPS-stimulated BV2 and murine primary microglial cells (parameters assessed: cell viability, intracellular ROS, levels of pro-inflammatory mediators, activation of signalling pathways)	[364]
**Diphlorethohydroxycarmalol**	0.5–50 µM	*I. okamurae*	Antioxidant	In vitro	HT22 cells treated by H_2_O_2_ (parameters assessed: cell viability, apoptosis, intracellular ROS, lipid peroxidation inhibitory activity, and intracellular Ca^2+^ level)	[367]
Different extracts containing phloroglucinol and other phenolic compounds		*H. elongata*	Antioxidant	In vitro	DPPH• scavenging activity	[369]

Olasehinde et al. [370] analysed extracts rich in phlorotannins, phenolic acid, and flavonoids from four seaweeds: *U. lactuca*, *Ecklonia maxima* (Osbeck) Papenfuss, *G. gracilis* and *G. pristoides*. The authors reported that the seaweed extracts attenuated the zinc-induced neurotoxicity in HT-22 cells by displaying antioxidant effects, reducing apoptosis, and inhibiting AChE.

The existence of flavonoids in algae is very controversial. There is unanimity in the claim that flavonoids are not expected to be found in algae due to the lack of enzymes in the biosynthetic pathway of these compounds, being considered unique to land plants, but there are more and more studies reporting the presence of flavonoids in algae. Although genes for the initial stages of the phenylpropanoid pathway are present in the genomes of Rhodophyta and Chlorophyta, there is still no evidence for the existence of specific flavonoid genes. On the other hand, the reported concentrations of flavonoid compounds in algae are less than those found in terrestrial plants [371,372]. The neuroprotective effect of a flavonoid-rich fraction obtained from *Ascophyllum nodosum* (L.) Le Jolis was evaluated in an Aβ_42_-expressing AD model of *Drosophila melanogaster*. At 2 mg/mL, this fraction had a strong effect on cell apoptosis, viability, longevity, mitochondrial dysfunction, and oxidative stress. At 2 mg/mL, the levels of H_2_O_2_, TBARS, and SOD activity were similar to those of the control group [373].

Epicatechin is the most abundant phenolic compound in macroalgae such as *L. japonica*, *E. bicyclis*, *U. pinnatifida*, *P. tenera*, and *P. palmata* [374]. Antioxidant activity is one of the major biological activities of this compound because it can scavenge ROS and modulate pathways such as Erk and Nrf2. The presence of ortho-hydroxyl groups, such as in ortho-catechol moiety in (-)-epicatechin, was identified as essential for the direct detoxifying effects in the reaction with O_2_^●−^ and H_2_O_2_ [375]. Epicatechin decreased ROS levels, increased GSH content, and inhibited mitochondrial swelling in the homocysteine-induced mitochondrial damage model [376]. Another study pointed out that epicatechin has anti-inflammatory properties by preventing the increase of TNF-α, iNOS, and NF-κB expression in a rat model treated with doxorubicin [377].

### 3.6. Other Compounds

Fucosterol, a sterol found in brown algae, isolated from *Padina australis* var. *cuneata* Tak. Tanaka & K. Nozawa, at 112 μM, efficiently inhibited AChE from *Electrophorus electricus* and BChE from equine serum. Besides that, fucosterol reduced the release of pro-inflammatory mediators such as IL-6 at concentrations ranging from 12 to 192 μM. The inhibition of IL-1β, TNF-α, and PGE2 occurred at concentrations greater than 48 μM in the C8-B4 microglia cell line treated with LPS [378]. Fucosterol acts on the CREB and the TrkB, suggesting that fucosterol maintains cell survival and functions as BDNF-mimetic [379]. CREB can act as a transcription factor for the production of BDNF [380]. As mentioned before, BDNF controls neuronal development and survival, and the TrkB is the receptor of BDNF. Fucosterol (100 µM) from *Sargassum horridum* Setchell & N. L. Gardner prevents Aβ_1–42_ oligomerization; the in silico studies pointed out that these inhibitions occur by destabilization of the antiparallel β-strand interactions [381]. Pre-treatment of fucosterol (10 µM) isolated from *E. stolonifera* revealed neuroprotective effects against soluble Aβ_1–42_ in primary hippocampal neurons and in ageing rats by decreasing the intracellular Ca^2+^ levels, upregulating BDNF-TrkB-ERK1/2 signalling, and preventing ER stress [382].

Silva et al. [383] isolated eleganolone, eleganonal, and fucosterol from *Bifurcaria bifurcata* R.Ross. The in vitro neuroprotective effects were evaluated on 6-OHDA-treated SH-SY5Y cells (PD model), and the anti-inflammatory potential was studied using LPS-stimulated RAW 264.7 macrophages. Among the three compounds, eleganolone (0.1–1 µM; 24 h) counteracted the neurotoxicity induced by 6-OHDA in about 20%, an effect due to its protection against mitochondrial disruption, oxidative stress, inflammation, and apoptosis, as well as its ability to inhibit the NF-kB pathway.

Glycolipids in methanol extract from a cultivated *Chondrus crispus* Stackhouse comprise monogalactosyl diacylglycerols and digalactosyldiacylglycerols. A fraction obtained from the methanolic extract of *C. crispus* enriched in monogalactosyl diacylglycerols significantly delayed the onset of Aβ_1–42_-induced paralysis in *C. elegans*. The effect was mediated by activating antioxidant defences [384]. Similarly, the methanolic extract from a cultivated *C. crispus*, at 0.5 mg/mL, showed antioxidant activity by upregulation of sod-3 and skn-1 genes and reduced the α-synuclein aggregation in the transgenic NL5901 strain of *C. elegans* [385]. SKN-1 is the *C. elegans* functional ortholog of the mammalian Nrf transcription factor and plays a crucial role against oxidative stress [386].

α-tocopherol (0.82 mM), as well as β-carotene (0.016 mM), in combination with ascorbic acid (1.6 mM), forming a vitamin complex, can promote the antioxidant effects by decreasing the ROS levels. Moreover, α-tocopherol (0.04 mM), β-carotene (0.0008 mM), and ascorbic acid (0.08 mM) promoted anti-inflammatory effects by increasing IL-4 and decreasing the IL-6 in peripheral blood mononuclear cells of AD patients [387]. On the other hand, a recent study concluded that, in a situation related to acute ischemic stroke, the excessive intake of α-tocopherol might increase microglial activation, and thus, the expression of pro-inflammatory cytokines [388].

Ursolic acid, a terpenoid compound, at 100 and 150 mg/kg, attenuated the traumatic brain injury in mice by activating the Nrf2-ARE signalling pathway, preventing oxidative stress and inflammatory process [389]. Another study reported that the administration of ursolic acid at 25 mg/kg body weight for 21 days effectively reduced the oxidative stress in a mouse model of PD induced by MPTP [390].

Racemosins A and B are alkaloids extracted from *Caulerpa racemosa* (Forsskål) J. Agardh that attenuated the toxicity in SH-SY5Y induced by Aβ25–35. In addition, racemosin A showed increased cell viability at 14.6% at 10 μM, more effective than racemosin B at the same concentration [391].

## 4. Gastrointestinal Digestion, Absorption, and Bioavailability of Algae-Derived Compounds

As mentioned before, seaweed-derived compounds have a great potential to exert neuroprotective activity. However, these compounds’ effectiveness depends on several parameters, such as ADME (absorption, distribution, metabolization, and elimination) and their ability to cross the BBB, as recently reviewed by Shikov et al. [392]. This review summarizes the pharmacokinetic studies of marine drugs isolated from different marine organisms (macroalgae, crustacean, sea cucumber, sea fungus, sea urchin, and other organisms). Concerning seaweed compounds, this review offers excellent information about pharmacokinetic parameters for fucoidan, griffithsin, alginates, halomon, eckol, fucoxanthin, and astaxanthin.

Only part of the compounds ingested in the food passes from the digestive system into the bloodstream. Bioavailability refers to this fraction of compounds absorbed, distributed by the circulatory system, and subjected to metabolism and elimination. On the other hand, bioaccessibility is the fraction of compounds released from the food matrix during digestion and becomes available for small intestinal absorption [393,394].

Polysaccharides from macroalgae have β (1→4) linkages that humans cannot digest, being indigestible dietary fibres. These fibres are partially fermented by colonic microflora into short-chain fatty acids (SCFA) [395,396]. However, fucoidan and ulvan are soluble dietary fibres, although they have high viscosity in an aqueous medium [396]. The fucoidan structure isolated from macroalgae belonging to the order Fucales is mainly composed of alternating (1→3) and (1→4)-linked α-L-fucose residues. On the other hand, the sulfated galactofucan found in brown seaweed species of the order Fucales are mainly composed of (1→6)-β-D-galactose and/or (1→2)-β-D-mannose units [397].

Due to the high molecular weight of fucoidan, this polysaccharide cannot be degraded by human digestive enzymes [396]. Chen et al. [395] reported that SPs extracted from *A. nodosum* were not degraded by human saliva because these SPs have α (1→6) linkages that are not degraded by salivary amylase. However, they also do not suffer degradation by artificial gastric and small intestinal juices in simulated digestion models. Only fermentation by the gut microbiota revealed degraded SPs.

Relatively to fucoidan in the brain, Rouzet et al. [398], using radiolabeled fucoidan with 99 mTc, reported low polysaccharide levels in the brain.

Pozharitskaya et al. [399] evaluated the pharmacokinetics and tissue distribution of fucoidan isolated from *F. vesiculosus* in rats after a single-dose oral administration (100 mg/kg). Pharmacokinetic parameters were recorded from plasma and tissues (liver, kidneys, spleen, striated muscle, and omentum). The highest concentration of fucoidan was found in the kidneys (Cmax = 1.23 µg/g), while the lowest level was measured in the plasma (Cmax = 0.125 µg/mL).

Protein digestion occurs in the gastrointestinal tract, where these macromolecules are broken by pepsins and pancreatic proteases that hydrolyse peptide linkages producing oligopeptides that, in turn, suffer additional cleavage by peptidases, being the resultant amino acids transported across the basolateral membrane and enter the portal circulation. In addition to free amino acids, dipeptides and tripeptides can cross the intestinal wall and enter the portal circulation [400]. Some di and tripeptides can be absorbed intact across the intestinal membrane [401]. Recently, it was also reported that some dipeptides are transported across BBB by different transport mechanisms [402].

α-linolenic acid is the precursor of ω-3 PUFAs such as EPA and DHA [403]. The human brain’s capacity for the biosynthesis of longer chain ω-3 fatty acids from its precursor ALA is very small. It is estimated that less than 1% of ALA consumed is converted into DHA in the brain, and this conversion occurs mainly in the liver [404].

PUFAs can be esterified into phospholipids, incorporated into high-density lipoprotein, and later transported to the liver. Likewise, DHA can be delivered into the brain in nonesterified plasma DHA and lipoprotein derived 2-DHA lysophospholipids [405].

Recently, Francisco et al. [406] studied the bioaccessibility of fatty acids, polyphenols, and the antioxidant activity before and after *Fucus spiralis* L. by an in vitro digestion model as a simulation of the human digestive system process. In fatty acids, it was concluded that the total lipid bioaccessibility was 12.1%, with EPA being the major ω-3 PUFA, present with a bioaccessibility of 13.0%. Meanwhile, the bioaccessibility of the antioxidants varied between 42.7% and 59.5%. However, the bioaccessibility of polyphenols in freeze-dried samples was lower (23.0%) [406].

In the small intestine, after emulsification with fats, carotenoids are incorporated into lipid micelles for absorption by intestinal enterocytes. Here, carotenoids are packaged into chylomicrons that enter the lymphatic system for delivery to the liver. Some carotenoids can be stored in this organ while the rest are repackaged into lipoproteins and released into the bloodstream. In the bloodstream, xanthophylls are carried in HDL cholesterol and carotenes in LDL cholesterol. Some carotenoids may be uptaken by peripheral tissues [407].

Due to lipophilicity, most carotenoids can cross the BBB. α-Carotene, α-cryptoxanthin, β-carotene, β-cryptoxanthin, lutein, lycopene, and zeaxanthin are examples of carotenoids found in the brain [332,333]. The xanthophylls (lutein, zeaxanthin, and cryptoxanthin) represent 72% of the total carotenoids in the brain. The majority corresponds to lutein with 34% [408].

Polyphenols suffer biotransformation and conjugation in the gastrointestinal tract, liver, and cells. These compounds can be conjugated into glucuronide, sulfate, and methyl groups in the gut mucosa and inner tissues, and the absorption occurs in the small intestine [409].

Corona et al. [410], using an in vitro digestion/fermentation method, studied the gastrointestinal digestion of phlorotannin-rich extracts from *A. nodosum*, having reported that compounds with molecular weight > 10 KDa were only slightly affected by the gastrointestinal digestion; however, these phenolic compounds were degraded mainly by the colonic fermentation in the large intestine. Additionally, the authors reported that the LC-MS/MS analysis of the gastrointestinal digestion and colonic fermentation extracts indicated the presence of some phlorotannins, such as hydroxytrifuhalol A, diphloretol/difucol, and 7-hydroxyeckol. Furthermore, the in vitro assays for testing antioxidant scavenging activity revealed a reduction of bioactivity after gastrointestinal digestion and colonic fermentation.

The BBB has characteristics that can hinder the passage of some molecules. Size and lipid solubility are important factors to consider. Several strategies have been developed to allow the efficient passage of compounds with neuroprotective activity through the BBB. Lipid carriers or liposomes, colloidal drug carriers, micelles, and β-cyclodextrin carriers are examples of these strategies [411]. These nanocarriers can either be functionalized with a different molecule of interest, such as transferrin or ApoE, to facilitate BBB transport. In addition, the transport of nanocarriers through the BBB can be increased by modification of their surface with cell-penetrating peptides (CPPs). Trans-activating transcription (TAT) peptide is an example of CPP that promotes the transport of nanoparticles across the BBB [412,413,414].

The development of nanoparticles itself is a challenge, being necessary to guarantee the stability of the nanoparticle during gastrointestinal digestion and its need to pass through the intestinal epithelium [415,416].

## 5. Macroalgae as Potential Prebiotics and the Gut–Brain Axis

As mentioned in the previous section, some compounds are considered non-digestible, as is the case of polysaccharides and phlorotannins, and need to be fermented by microflora. Over time some definitions of the term prebiotic have been proposed. Generally, prebiotic refers to non-digestible compounds that, when undergoing metabolism by the gut microbiota, modulate the composition and/or activity of the microbiota, benefiting the host [417]. The interest in prebiotics is increasing. The composition of the digestive microbiota, the microbial community in the digestive tract, can affect human health by supplying nutrients, converting metabolites, and acting as a barrier to pathogenic bacteria [418,419,420].

The gut microbiota consists mainly of anaerobic bacteria, comprising approximately 500–1000 species. Firmicutes and Bacteriodetes are the most abundant phyla; bacteria of the phyla Proteobacteria, Verrumicrobia, Actinobacteria, Fusobacteria, and Cyanobacteria are present in human gut microbiota [421].

Many studies have suggested a role for the gut microbiota in the gut–brain interactions, pointing out that neurodegenerative diseases can be associated with gut microbiota, modulating the response to stress and neuroinflammation [422,423,424].

Studies using germ-free (GF), or specific pathogen-free (SPF), rodents showed that the gut microbiota influences the development of emotional behaviour, stress modulation, and brain neurotransmitter systems. Specific pathogen-free (SPF) BALB/c mice treated with oral antimicrobials revealed an increased hippocampal expression of BDNF [425]. In male GF Swiss Webster mice, a significant elevation in the hippocampal serotonin concentration and the increased concentration in plasma of tryptophan, the precursor of serotonin, indicates that microbiota can influence CNS serotonergic neurotransmission. Additionally, in this study, a decrease in BDNF expression in the hippocampus was reported only in male animal models [426]. Neufeld et al. [427] reported that in GF female Swiss Webster mice, the levels of BDNF were increased in the hippocampus. Besides that, a decrease in NMDA receptor NR2B mRNA expression in the central amygdala was reported, as well as a decrease in serotonin receptor 1A (5HT1A) mRNA expression in the dentate gyrus.

In macroalgae, some compounds have prebiotic effects. For example, Laminaran obtained from *Sargassum crassifolium* J. Agardh, a non-digestible oligosaccharide, can act as a prebiotic by increasing the cell biomass of *Lactobacillus plantarum* FNCC 0051 (Firmicute) and *Bifidobacterium longum* FNCC 1081 (Actinobacteria) [129]. Fucoidan extracted from *Fucus evanescens* C. Agardh is non-digestible in the upper gastrointestinal tract and stimulates *Bifidobacterium bifidum* growth in vitro [428]. Chen et al. [395] investigated the effects of *A. nodosum* polysaccharides on gut microbiota composition by bacterial 16S ribosomal RNA sequencing using faecal fermentation samples. They reported that these polysaccharides favoured the growth of the Bacteroidetes, especially *Bacteroides ovatus*, and Firmicutes, increasing the SCFA content after fermentation. Another study using in vitro fermentation of 0.8 g of fucoidan from *L. japonica* by human faecal microbiota, during 48 h, increased *Lactobacillus* and *Bifidobacterium*, and the SCFA, including acetic, butyric, and lactic acids, also increased [429].

*Kappaphycus alvarezii* (Doty) L. M. Liao, a source of к-carrageenan, was submitted to an in vitro model of digestion and fermentation. At 1% (*w/v*), after 24 h, *Bifidobacterium* sp. growth was increased, and *Clostridium coccoides* and *Eubacterium rectale* decreased. Total SCFA production, especially acetate and propionate, increased [430].

Watson et al. [431] reported that supplementation of 4 g/day of mixed EPA/DHA over eight weeks in healthy middle-aged individuals increased the SCFA-producing bacteria, especially butyrate-producing bacterial genera (Firmicutes) as *Lactobacillus*, *Lachnospira*, and *Roseburia*. In another study, the supplementation with 350 mg/day of DHA in middle-aged and older women increased the Lachnospiraceae family [432].

The phlorotannin-enriched fraction from *Ecklonia radiata* (C. Agardh) J. Agardh suffering in vitro anaerobic fermentation by human faecal inocula increased *Faecalibacterium prausnitzii* and decreased *Enterococcus* number. The phlorotannin-enriched fraction can contribute to partially inhibiting pathogenic bacterial growth and preventing inflammatory diseases of the mammalian intestine [433].

Honarpisheh et al. [434] reported that Aβ deposition in the brain occurs after inflammation by dysregulated gut homeostasis in Tg2576 mice, having dysfunction of the intestinal epithelial barrier, and vascular Aβ deposition in the intestinal epithelial barrier before cerebral Aβ aggregation.

In one study, the faecal microbiome collected from 72 PD patients and age-matched controls revealed that the abundance of Prevotellaceae in faeces of PD patients was decreased, and the content of Enterobacteriaceae was increased. *Prevotella* present in the colon can degrade complex polysaccharides, providing SCFA with neuroprotective value. Besides that, reducing Prevotellaceae family abundance is related to increased gut permeability. The Enterobacteriaceae are related to the severity of postural instability and gait difficulty [435].

Peng et al. [436] reported alterations in gut microbiota composition in a senescence-accelerated mouse prone 8 (SAMP8) (AD model) using both 16S rRNA gene and metagenomics sequencing of faecal samples. These alterations corresponded to a decrease in the relative abundance of the Bacteroidales and increased content of Lachnospiraceae, Alistipes (family Rikenellaceae), and Odoribacter (family Odoribacteraceae). This set of changes in microbiota affected the transport and metabolism of inorganic ions, coenzymes, nucleotides, lipids, and energy production and conversion. For example, the genus norank_f__Bacteroidales_S24-7_group also decreased in SAMP8 mice, which plays an important role in electron transport [436].

In G93A transgenic mice, as a model of human ALS, the intestinal microbiome is changed and damaged tight junctions were observed before ALS disease onset. After administration of 2% butyrate, an SCFA, in the filtered drinking water, for 2.5 months, an increase in the abundance of the butyrate-producing bacteria *Butyrivibrio* spp., especially *Butyrivibrio fibrisolvens* and *Clostridium* and *Ruminococcus* spp. was observed, correcting the dysbiosis in this animal model [437].

The comparison between the intestinal microbial of healthy people and ALS patients by high-throughput sequencing technology revealed the decreased abundance at the genus level of Oscillibacter, Anaerostipes, and Lachnospiraceae and increased at the genus level of Dorea. Anaerostipes plays an important role in converting lactate into butyrate, and Lachnospiraceae produces butyric acid [438].

Wang et al. [258] suggested a potential mechanistic link between gut microbiota dysbiosis and neuroinflammation in AD progression. Using AD mouse models, it was discovered that the alteration of gut microbiota leads to the peripheral accumulation of phenylalanine and isoleucine, which stimulates the differentiation and proliferation of pro-inflammatory T helper 1 (Th1) cells during AD progression. The brain-infiltrated peripheral Th1 immune cells are associated with the M1 microglia activation, contributing to AD-associated neuroinflammation. The elevation of phenylalanine and isoleucine concentrations and the increase of Th1 cell frequency in the blood were also observed in patients with mild cognitive impairment (MCI) related to AD. Recently, sodium oligomannate demonstrated cognition improvement in a phase III clinical trial in China, suppressing gut dysbiosis and the associated phenylalanine/isoleucine accumulation, harnessing neuroinflammation, and reversing cognition impairment. These findings show the role of gut dysbiosis-promoted neuroinflammation in AD progression while suggesting a novel strategy for AD therapy by modulating the gut microbiota.

Nowadays, limited information from human studies is available, possibly by the complexity of studying the human microbiota, which is affected by several conditions, including diet, sex-related differences, and genetic variation, among other aspects.

## 6. Clinical Studies

Clinical trials with pure algal metabolites are scarce, with the majority aimed to ascertain the effect of algae consumption, as extracts or fractions, on cancer, obesity, and diabetes [9]. The clinical trials regarding neuroprotection or cognitive studies were selected from the literature, ClinicalTrials.org, and Cochrane library and were related to isolated compounds or extracts.

One study involved nine patients with severe traumatic brain injury and traumatically induced coma presenting a Glasgow Coma Scale [GCS] score ≤ 8 after administration of a nutritional supplement of 16.2 g of purified ω-3 PUFAs oil (EPA:DHA of 2:1) delivered daily. Supplementation increased the GCS score in all patients. However, this study has some limitations, recognized by the authors, such as a small sample size, nonrandomized design, and a placebo absence [439]. Moreover, EPA and DHA are ω-3 PUFAs that play an essential role in preventing and treating psychiatric disorders [440]. However, one study reported that individuals with AD that received 2 g/day of algal DHA for 18 months revealed no beneficial effects compared to the placebo group in slowing the rate of cognitive decline, assessed by the rate of change on the cognitive subscale of the AD Assessment Scale (ADAS-cog score) and change in the Clinical Dementia Rating (CDR) sum of boxes [441]. DHA can exert neuroprotective action against neurodegenerative diseases and cerebrovascular diseases, especially in the injury produced by ischemia-reperfusion [442]. DHA is the most abundant in the CNS, mainly in the prefrontal cortex and the hippocampus [291]. This fatty acid is present in large amounts in neuron membranes in cortical grey matter. Therefore, it plays an important role because membrane status is closely related to neuronal information transfer, signal transduction speed, and interaction with proteins [443]. DHA has an important role, particularly in fetal development, promoting neuronal development, and synaptic plasticity [444]. Furthermore, DHA can upregulate the Gpx4 gene, stimulating the mechanisms to protect from oxidative stress [445]. One study, performed in rhesus monkeys (*Macaca mulatta*) using noninvasive resting-state functional connectivity MRI, demonstrated that deficiency diet in ω-3 PUFAs, especially DHA, harms large-scale brain organization and visual pathway connectivity [446].

Homotaurine has a neuroprotective effect and has been studied as a possible therapeutic agent for AD. In a Phase III clinical trial, this compound, however, was not effective as a potential treatment [14]. The limitations of this study were the lack of adequate statistical validity of the analysis models, the diversity of the disease, and the impact of the mixing of effects of the demographic and clinical variables. Consequently, the authors developed revised predictive post-hoc models that showed positive and significant effects on secondary endpoints and subgroups of patients with a trend toward a treatment effect for ADAS-cog but with no slowing of decline in the CDR-SB. It also showed significantly less hippocampal volume loss when using 100 mg and 150 mg of homotaurine, compared to placebo and a reduction in global cognitive decline in memory and in APOE4 allele carriers suggesting that this compound has positive effects on AD [14]. Another study on cognitive impairment from 2018 presented positive benefits of homotaurine [447]. This study aimed to evaluate the effects of one-year administration of 100 mg total dose homotaurine (tramiprosate)/day in 245 patients from 28 different centres in Italy presenting mild symptoms of cognitive impairment. Mini Mental State Examination (MMSE) was used to evaluate the evolution of the cognitive decline over time. The authors reported significant improvements in patients at months 4, 8, and 12 depending on the gravity of the decline, concluding that homotaurine may be considered a potential symptomatic treatment for cognitive functions. However, it is necessary to assess whether this compound could affect the progression of cognitive decline [447]. Recently, it was reported that homotaurine (as ALZ-801) is in a phase III study expecting FDA approval as the first disease-modifying drug for AD [448].

In another single blind, randomized, controlled study, homotaurine (100 mg) safety and efficacy was evaluated in patients with PD and cognitive impairment against a group with no homotaurine administration. Patients were evaluated at baseline and after 6 months, including motor and non-motor functions and decline (Unified PD Rating Scale, UPDRS), disability and quality of life, depression, excessive daytime sleepiness, and tiredness. Extensive neuropsychological tests were performed to evaluate specific cognitive domains: memory, phonemic verbal fluency, executive functions, and selective visual attention. Forty-seven patients were evaluated at baseline, and 24 (51 %) completed the study (PD-homotaurine: 44% and PD-controls: 59%). Intention to treat analyses to evaluate homotaurine safety showed mild side effects (gastrointestinal upsetting) in three patients. Homotaurine efficacy showed no difference between groups, but within-group analyses of PD-homotaurine patients had a better score at UPDRS-I after 6 months compared to baseline on the Epworth Sleepiness Scale. No significant difference was seen in the PD-control group. The authors concluded that homotaurine is a safe drug and has a probable beneficial effect on excessive sleepiness. However, more studies are necessary to confirm this potential effect in promoting the sleep/awake cycle in PD patients [449].

Fermented *L. japonica*, which shows neuroprotective and antioxidant activities, was used to assess if this fermented seaweed may be considered a potential supplement that can be administered to elderly people to reduce neurodegenerative conditions associated with ageing. Forty senior subjects participated in a randomized, double-blind, and placebo-controlled study forming two groups, one treated with 1.5 g/day of fermented seaweeds for six weeks and the other with a placebo (control group). To evaluate short-term memory, neuropsychological tests were used. Body composition, physical evaluations, antioxidant, and inflammatory markers were also assessed in the analyses pre- and post-test [450]. The results showed that the fermented seaweed significantly improved neuropsychological test scores, including higher scores in the K-MMSE, numerical memory test, Raven test, and iconic memory, compared to the control group; shorter test trial times in the 6-min walk test were reported and also significantly increased antioxidant activity and lowered 8-oxoDG levels. The authors concluded that this fermented seaweed might provide a protective mechanism against cognitive impairment associated with dementia, also preserving physical function in the elderly and improving antioxidant activity that can act against progressive degeneration caused by ROS [450].

Sodium oligomannate, with neuroprotection activity against AD, was assessed by a phase II trial, during a 24-week treatment, against a placebo control. Patients received 900 mg or 600 mg of sodium oligomannate or a placebo capsule during the treatment period. The primary outcome was assessed by changes in ADAS-cog12 scores from baseline to week 24. Efficacy outcomes included CIBIC-Plus, ADCS-ADL, and NPI at 24 weeks after treatment compared with baseline. The authors reported that compared with the placebo group, the ADAS-cog12 score changed in the 600 mg group to −1.39, and the 900 mg group was −2.58. The CIBIC-Plus assessment was significantly higher in the 900 mg group than the placebo group. Furthermore, the 900 mg subgroup showed a lower decline in cerebral metabolic rate for glucose than the placebo subgroup at the left precuneus, right posterior cingulate, bilateral hippocampus, and bilateral inferior orbital frontal. The authors concluded that this compound was safe and well tolerated, and 900 mg was chosen for further studies [15]. The phase III clinical trial was conducted in participants with mild-to-moderate AD to assess sodium oligomannate efficacy and safety. Participants (818) were randomized to placebo (410) or 900 mg of the compound (408) for 36 weeks. The authors assessed the drug-placebo difference from baseline on the ADAS-cog12, Clinician’s Interview-Based Impression of Change with caregiver input (CIBIC^+^), AD Cooperative Study-Activities of Daily Living (ADCS-ADL) scale, and Neuropsychiatric Inventory (NPI). A significant drug-placebo difference on the ADAS-Cog12, favouring sodium oligomannate, was reported at each measurement time point after week 4 and continuing throughout the trial. The difference between the groups in change from baseline was −2.15 points after 36 weeks of treatment, demonstrating significant improvement in cognition across all observation periods of the 36-week trial. The compound was safe and well-tolerated by all patients [451].

Phlorotannins present in brown seaweeds have been shown to inhibit α-amylase and α-glucosidase, important enzymes involved in the degradation and intestinal absorption of carbohydrates [452]. Closely related to this are the modulation of post-prandial glycemic response in mice and increased insulin sensitivity in humans after supplementation with seaweed extract. Haskell-Ramsay et al. [452] explored the effect of brown seaweed extract on post-prandial cognitive function in 60 healthy adults divided into two groups, with the control groups being supplemented with a placebo. Episodic memory, attention, and subjective state were assessed at baseline and five times at 40 min intervals over a 3 h period following lunch, with either extract or placebo consumed 30 min before lunch. Seaweed extracts significantly improved the accuracy in tasks such as digit vigilance and choice reaction time. These results show the potential modulation of cognition with seaweed extract, but the authors suggest future studies using blood sugar and insulin responses to assess the mechanism connected with these effects [452].

In another study, it was hypothesized that phlorotannin supplement could improve sleep in patients with self-reported sleeping problems with impact on cognitive performance. In a randomized, double-blind, placebo-controlled trial, 24 subjects consumed either a placebo or 500 mg/day phlorotannin supplement for 1 week, 30 to 60 min before bedtime. Sleep parameters were assessed at baseline and at every week with sleep questionnaires and polysomnography. The authors reported that phlorotannin supplement significantly increased the “Sleep duration” scores compared to the placebo, but there were no significant differences in the total Pittsburgh Sleep Quality Index (PSQI) scores. Polysomnography revealed that wakefulness after sleep onset and total wake time was significantly lower in the phlorotannin group compared to the placebo group. Additionally, the respiratory disturbance index during supine rapid eye movement sleep was significantly lower in the phlorotannin group. The authors concluded that the phlorotannin supplement can improve sleep maintenance [453].

## 7. Safety of Consumption of Seaweeds

Seaweeds are an excellent nutritional source for their wide variety of nutrients and their biological activities, especially in neuroprotection.

Macroalgae have essential elements for human nutrition and health, such as Ca^2+^ and magnesium, mainly found in high amounts in red and green macroalgae, as well as toxic elements such as heavy metals, pesticides, radioactive isotopes, dioxins, and others [269,454].

Seaweeds can accumulate various heavy metals from water or sediments, and the presence of these elements in marine environments is mainly a result of anthropogenic activities [455]. The absorption capacity of heavy metals by macroalgae is related to polysaccharides present in the cell wall since they can bind to heavy metals, with a greater affinity observed in brown algae [456].

Desideri et al. [457] evaluated the heavy metal (aluminium, arsenic, nickel, cadmium, and lead) bioaccessibility from seaweeds by the in vitro gastrointestinal digestion method and reported that bioaccessibility is very high for cadmium and poor for aluminium and lead.

Cadmium can be found in *Alaria esculenta* (L.) Greville, *Laminaria digitata* (Hudson) J.V. Lamouroux, *L. japonica*, *U. pinnatifida*, and others. The maximum limit varies with legislation among different countries. For example, in Australia and New Zealand, it is 0.2 mg/kg dw, while in Europe, it is 3.0 mg/kg. Some species, such as *L. japonica,* surpass the limit for this heavy metal content for human consumption [458,459,460].

Arsenic is mainly accumulated in Ochrophyta species, where some species, such as *Sargassum piluliferum* (Turner) C. Agardh, can accumulate over 100 mg/kg dw of inorganic As [461]. The regulation of the maximum limit of inorganic arsenic allowed varies between countries, with the highest value (3.0 mg/kg) allowed in France and USA [459]. *L. digitata* is one of the species with a higher capacity for accumulating arsenic, and it can be found in the form of inorganic arsenic, arsenosugars, and arsenolipids [462].

An analysis of 52 samples from 11 algae-based products revealed that lead and mercury content did not exceed the maximum level of French recommendations, 5 mg/kg dw and 0.1 mg/kg dw, respectively [454,463].

Iodine is essential for producing thyroid hormones—triiodothyronine and thyroxine. However, excessive iodine consumption can dysregulate levels of these hormones [464]. The tolerable upper intake level recommended by the Scientific Committee for Food for iodine is 600 μg/day. The French recommendations point to 2000 mg/kg dw of maximum level in seaweed [454]. The iodine content in *L. digitata* can be over 7000 mg/kg dw [454,465].

Polycyclic aromatic hydrocarbons (PAHs) and polychlorinated biphenyls (PCBs) are chemical pollutants that firstly result from incomplete combustion of fossil fuels, garbage incinerators, vehicle engines, and secondly as the result of leakages from electrical transformers, wastes disposal, and spillage [466]. Fogaça et al. [467] reported that *Ulva* spp. contain concentrations of PAHs up to 51.8 µg/kg in dried samples. However, considering the culinary treatment and bioaccessibility of PAHs, the authors concluded that it was safe for consumption. In another study, the level of PCBs reported in *U. lactuca* was 7 µg/kg dw, as the levels of PCBs are related to levels in sediments [468].

Radionuclides have high nuclear energy, thus becoming unstable [469]. Prolonged exposure to radionuclides over the recommended limit may contribute to the appearance of cancer and metabolic disorders [470,471]. Technetium-99 is one example of radionuclides found in brown algae, such as *F. vesiculosus* and *A. nodosum*; the values of radionuclides may vary from season to season [472]. *C. crispus* can accumulate high values of lead-210 and polonium-210, *P. palmata* has a high affinity to accumulate potassium-40, and *U. lactuca* accumulates radium-230, thorium-230, and thorium-232 [473].

Additionally, several toxins are found in macroalgae, such as macrolide compounds, including polycavernoside A, aplysiatoxin, debromoaplysiatoxin, and manauealide A, B, and C. However, these toxins are believed to originate from cyanobacteria [474]. For example, polycavernoside A, a macrolide compound found in red algae *Gracilaria edulis* (S. G. Gmelin) P. C. Silva, may provoke gastrointestinal disturbances and neurological alterations [475,476]. Another red macroalga, *G. vermiculophylla*, produces PGE2 from AA and may cause gastrointestinal disturbances [477]. Manauealide A, B, and C are present in *Gracilaria coronopifolia* J. Agardh and G. edulis [478]. Debromoaplysiatoxin and Aplysiatoxin are other toxins present in *G. coronopifolia* [479].

## 8. Conclusions and Future Prospective

This review provides evidence that macroalgae are rich sources of bioactive compounds that exhibit neuroprotective effects with potential therapeutic effects for managing and preventing neurodegenerative disease. Numerous compounds such as those isolated from macroalgae have been shown to exert neuroprotective effects through several mechanisms.

Phlorotannins, SPs, and carotenoids isolated from different macroalgae species showed antioxidant and anti-inflammatory activities by regulating several pathways. The inhibition of BACE1, AChE, and BChE, in AD, hMAO-B in PD, and MAO-A in depression was performed efficiently by SPs, glycoproteins, carotenoids, especially fucoxanthin, and phlorotannins. The inhibition of Aβ aggregates can be achieved by SPs, fucoxanthin, and phlorotannins. The PUFAs are essential against neuroinflammation. The neuroprotective role of some of these classes of compounds and their potential safety even at higher doses have been confirmed recently in different clinical trials.

On the other hand, studies about the potential adverse effects of compounds derived from macroalgae are necessary. Studies on the structure-activity relationship of compounds derived from macroalgae will be necessary to develop drugs for the treatment of neurodegenerative diseases. Typically, in studies carried out in vitro or in animal models, the concentration of the compounds derived from macroalgae used is considerably higher than those which humans can access via food. Further research is needed to evaluate the potential adverse effects of human algae consumption, considering the different variables (e.g., processing, the toxicity of the chemical forms of the elements).

Given the growing interest in macroalgae in several areas, including the food and hydrocolloid industry, it is necessary to guarantee sustainable production. Moreover, the legislation on the content of specific components present in macroalgae is significantly different between countries in some cases, and there is still a need for legislation on which species are reliable for human consumption. It is essential to encourage weighted consumption and the purchase of safe algae from a food standpoint.

## Figures and Tables

**Table 1:** IC_50_ value of cholinesterase, BACE1, and MAO inhibitions.

Compounds	IC_50_ Value	Reference
**Cholinesterases Inhibition**		
Sulfated polysaccharides extracted from *Ulva lactuca* L.	106.93 µg/mL (AChE)/93.45 µg/mL (BChE)	[203]
Glycoprotein isolated from *U. pinnatifida*	63.56 μg/mL (AChE)/99.03 μg/mL (BChE)	[204]
Fucoxanthin	81.2 µM (AChE)	[205]
Phlorotannin 974-B	1.95 µM (AChE)/3.26 μM (BChE)	[206]
α-Linolenic acid	12.50 μg/mL (AChE)/15.89 μg/mL (BChE)	[207]
**BACE 1 Inhibition**		
Glycoprotein isolated from *U. pinnatifida*	73.35 μg/mL	[204]
Fucoxanthin	5.31 μM	[208]
Fucosterol	64.12 μM	[208]
Dioxinodehydroeckol	5.35 μM	[209]
Eckol	12.20 μM	[209]
Phlorofurofucoeckol A	2.13 μM	[209]
Dieckol	2.21 μM	[209]
Triphloroethol A	11.68 μM	[209]
7-Phloroethol	8.59 μM	[209]
Fucofuroeckol-b	16.1 μM	[210]
**MAO Inhibition**		
Fucoxanthin	197.41 μM (MAO-A)/211.12 μM (MAO-B)	[211]
Eckol	7.20 μM (MAO-A)/83.44 μM (MAO-B)	[212,213]
Dieckol	11.43 μM (MAO-A)/43.42 μM (MAO-B)	[212,213]
Phlorofucofuroeckol-A	9.22 μM (MAO-A)/4.89 μM (MAO-B)	[213]

## Data Availability

Not applicable.
